# Understanding accommodative control in the clinic: Modeling latency and amplitude for uncorrected refractive error, presbyopia and cycloplegia

**DOI:** 10.1167/jov.24.3.4

**Published:** 2024-03-15

**Authors:** Jenny C. A. Read, Gerrit Maus, Clifton M. Schor

**Affiliations:** 1 Newcastle University, Faculty of Medical Sciences, Newcastle-upon-Tyne, UK; 2 Magic Leap Inc, Zurich, Switzerland; 3 UC Berkeley, Berkeley, California

**Keywords:** presbyopia, accommodation, control theory, computational modeling, refractive error

## Abstract

Accommodation is the process of adjusting the eye's optical power so as to focus at different distances. Uncorrected refractive error and/or functional presbyopia mean that sharp focus may not be achievable for some distances, so observers experience sustained defocus. Here, we identify a problem with current models of accommodative control: They predict excessive internal responses to stimuli outside accommodative range, leading to unrealistic adaptation effects. Specifically, after prolonged exposure to stimuli outside range, current models predict long latencies in the accommodative response to stimuli within range, as well as unrealistic dynamics and amplitudes of accommodative vergence innervation driven by the accommodative neural controller. These behaviors are not observed empirically. To solve this issue, we propose that the input to blur-driven accommodation is not retinal defocus, but correctable defocus. Predictive models of accommodative control already estimate demand from sensed defocus, using a realistic “virtual plant” to estimate accommodation. Correctable defocus can be obtained by restricting this demand to values physically attainable by the eye. If we further postulate that correctable defocus is computed using an idealized virtual plant that retains a young accommodative range, we can explain why accommodative–convergence responses are observed for stimuli that are too near—but not too far—to focus on. We model cycloplegia as a change in gain, and postulate a form of neural myopia to explain the additional relaxation of accommodation often seen with cycloplegia. This model produces plausible predictions for the accommodative response and accommodative convergence signal in a wide range of clinically relevant situations.

## Introduction

As we look around the world, we need to adjust our ocular accommodation to focus on objects at different distances. To achieve this, the brain controls contraction in the ciliary muscle and thus the optical power of the crystalline lens. Although this task is simple conceptually, it is complicated by issues such as sensorimotor latencies, the response properties of the muscle and lens, and the finite range of optical powers achievable by the lens. Formal models of this process have several benefits. Practically, they act as summaries of empirical findings, helping us to predict accommodative behavior to arbitrary stimuli and thus enabling professions from optometrists to visual display engineers to predict when people will experience blur. Models also represent theories about computations carried out by the brain, which can in principle be tested and compared with neurophysiological findings, and so help us to understand the nervous system.

**Figure 1.**
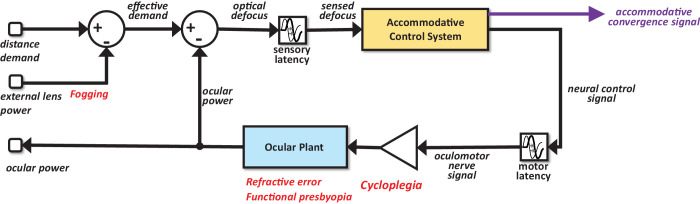
Overall model structure, representing accommodation as a negative feedback system. Red text shows where in the model four common clinical situations are represented. Demand owing to distance (measured in diopters) combines with any external lenses to produce an effective stimulus demand at the eye. The difference between this and ocular power (i.e., the optical power of the eye relative to the power needed to focus at infinity) gives the optical defocus. This in turn is sensed by the brain following a sensory latency allowing time for defocus to be computed. The accommodative control system takes the sensed defocus as input and outputs a neural control signal for accommodation. We also show the accommodative convergence signal (purple) which goes into the vergence control system, not considered in detail in this article. The neural control signal for accommodation travels down the oculomotor nerve and arrives at the ocular plant. We label the signal “neural control signal” when it is computed in the brain, and “oculomotor nerve signal” when it arrives at the plant after the motor latency. Cycloplegia, for example with atropine or cyclopentolate, effectively inhibits this signal, as indicated by the triangular gain block. In complete cycloplegia, gain is reduced to zero and effectively removes all input to the plant (i.e., cuts the signal). In partial cycloplegia, gain is reduced and a large steady-state defocus error manifests as a reduced amplitude of accommodation and elevated AC/A ratio. The ocular power is the output of the ocular plant. The internal structure of the blue Ocular Plant block is shown in Figure 2. Possible internal structures for the yellow Accommodative Control System block are discussed in Figures 4, 5, and 6. Figure 4 shows the structure we are proposing in this article.

As illustrated in Figure 1, models of accommodative control are essentially a negative feedback loop. The brain wishes to control ocular accommodation so as to make the retinal image as sharp as possible (which we shall here assume means minimizing defocus, although see Labhishetty, Cholewiak, Roorda, & Banks, 2021). This goal is achieved by sending a neural control signal down the oculomotor nerve to the ciliary muscle surrounding the ocular lens, that is the ocular “plant,” to ensure that the ocular power matches the effective optical demand of the stimulus. This neural control signal represents the ocular power requested by the brain, and thus can be represented in units of diopters (D). Much previous work suggests that the controller at the heart of this negative feedback loop, inside the golden block labelled “accommodative control system” in Figure 1, is essentially a leaky integrator. That is, the neural control signal is a weighted average of recent retinal defocus, with more recent defocus weighted more heavily.

We recently published a model of neural control of accommodation that aimed to bring together many ideas in the literature and to be reasonably comprehensive (Read et al., 2022). We also added features to account for accommodative micro-fluctuations and for the closed-loop resonance enhancing responses at 1 to 2 Hz. In particular, we emphasized the predictive nature of accommodative control: to avoid instability and ringing, given the relatively large sensorimotor latencies, the brain needs to take into account the future effect of the motor signal already sent to the ciliary muscle when considering how to respond to the current sensed defocus.

The model of Read et al. (2022) did not consider common clinical situations such as fogging lenses, cycloplegia, uncorrected (or imperfectly corrected) refractive error, and functional presbyopia. The last two factors in particular are very common in the general population, so models must make sensible predictions in these situations to be of practical use. The red text labels in Figure 1 show where these four clinical situations are represented in models. None of them directly affect the model of control, that is, the gold block labeled accommodative control system. However, as we explain elsewhere in this article, we find that our original model of control makes unrealistic predictions for these clinical situations. This indicates that, in fact, models of accommodative control have been incomplete, in a way that did not show up in the limited tests carried out to date. In this article, we suggest how to modify our understanding of accommodative control to obtain a useful model that gives sensible predictions both in standard situations and in common clinical situations such as functional presbyopia.

### Representing common clinical situations in models of accommodation

We first briefly explain how to represent these clinical situations within models (Figure 1) (see Methods for a more detailed discussion). In clinical optometry, “fogging” is the practice of adding plus lenses in front of the patient's eye so as to encourage accommodation to relax to the maximum amount possible (Ramsdale & Charman, 1988; Reese & Fry, 1941). This step is particularly important in patients with hyperopia who, because of their refractive error, will usually be accommodating even when viewing distant stimuli. The plus lenses effectively move the stimulus beyond the patient's far point, making it appear blurred or “fogged”, and any accommodation increases the defocus further. In models, fogging or other external lenses must simply be subtracted from the demand implied by the physical distance to produce an effective optical demand (Figure 1).

**Figure 2.**
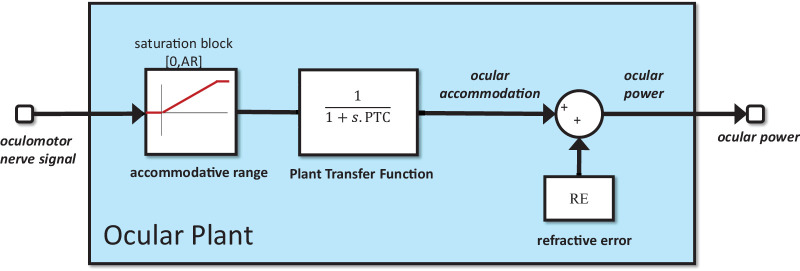
Structure of the ocular plant block shown in Figure 1. The basic transfer function is a leaky integrator or first-order low-pass temporal filter, with a time constant given by the model parameter PlantTimeConstant (abbreviated PTC; Table 1). This is followed by a saturation block representing the finite accommodative range, set by model parameter AccomRange, abbreviated AR. Inputs in the range [0, AR] are passed unchanged; inputs less than zero or greater than the AR produce outputs of zero or the AR, respectively. Functional presbyopia refers to the gradual reduction of the accommodative range as we age. Finally, the model parameter RefractiveError is added, abbreviated RE. For emmetropes, RE = 0; for myopes, RE > 0; for hyperopes, RE < 0.

We represent cycloplegia as a gain applied to the oculomotor nerve signal (van der Hoeve & Flieringa, 1924; Figure 1). With no cycloplegia, the gain is 1; for complete cycloplegia, the gain is 0. We represent refractive error as a constant offset added onto accommodation to produce ocular power (Figure 2). Under this definition, refractive error is the optical power of the eye under complete cycloplegia (see Methods). Finally, we represent the limited range of accommodation seen in functional presbyopia as a saturation block on the signal reaching the ocular plant (Figure 2).

### The problem: Integrator wind-up

These are standard representations of these clinical situations in models of accommodation, and we believe they are essentially correct. However, it has not previously been pointed out that under current leaky–integrator models of accommodative control, limiting the range of accommodation predicts a form of adaptation that is not observed. Current models predict that viewing a stimulus beyond the accommodative range should cause unrealistically long latencies in the accommodative responses to subsequent stimuli within the accommodative range. In control theory this phenomenon, occurring when an actuator cannot produce the commanded response, is known as integrator wind-up (Astrom & Rundqwist, 1989; Baheti, 1989; Fertik & Ross, 1967).

The predicted latencies stem from the very large internal signals predicted in response to sustained defocus. Consider for example the situation where an uncorrected +2 D myope views a stimulus at infinity. The correct ocular power would be 0 D, but the myope's elongated eyeball means that their ocular power is +2 D, even when the lens is fully relaxed. Therefore, they experience a steady optical defocus of −2 D (where optical defocus is defined as effective demand minus ocular power). Models of the neural control of accommodation are based on leaky–integral control, where the signal sent to the ocular lens is based on the integrated defocus encountered over the last few seconds. (In predictive models, the signal is actually based on the brain's own estimate of defocus, but we can neglect this distinction for the moment.) In this example, the leaky integrator thus asymptotes to a large negative value, equal to the integrator's gain multiplied by the steady-state defocus. The integrator's gain must be large, around 8, to keep defocus errors small for stimuli within the accommodative range (Read et al., 2022), and so its asymptotic value for stimuli beyond range is large, −16 D in this example. If the myope now transfers their gaze to a nearby object at +4 D, the defocus will change from −2 D to +2 D. However, the signal sent to the lens will remain negative until the leaky integrator has fully discharged from its asymptotic value of −16 D; only then will the lens begin to constrict as the signal begins to go positive. The response of accommodation to sinusoidal stimuli suggests a time-constant for the leaky integrator in the range of 2.5 seconds (Read et al., 2022). Thus, the latency before a response can be significant, well beyond the ∼300 ms corresponding with the sensorimotor latency. In fact, when we simulate this example (Figure 7i), we find a delay of more than 2 seconds before any response begins.

Although we have been unable to find published measurements of the latency of accommodative responses in uncorrected myopes, latencies measured in seconds are clearly unrealistic. They predict, for example, that a myopic child who has been trying to read the whiteboard several meters away at the front of the classroom would encounter a delay of some seconds before they could focus again on their own writing. Notice that the model is not simply predicting slower than usual responses: it is predicting a latency of some seconds before any response begins. This effect is also quite distinct from other forms of adaptation, such as nearwork-induced transient myopia (Hung & Ciuffreda, 2002, Hung & Ciuffreda, 1999). Current models make similarly unrealistic predictions for hyperopia and for functional presbyopia: in each case, exposure to a stimulus closer than the near point is predicted to cause large delays in the response to subsequent stimuli within range. Optometrists and people with refractive error can confirm that this is not their experience. Current models also predict very long (seconds) latencies after defocus owing to fogging. When the plus lenses are removed and the person looks at a nearby object, current models predict a delay of several seconds before the patient's focus begins to return to normal. Again, this adaptation to fogging is not seen empirically.

Evidently, if accommodative control can indeed be understood as a form of integrative control, the brain has developed its own form of an anti–wind-up mechanism to prevent the finite range of accommodation resulting in integrator wind-up and long latencies. Yet this factor is not accounted for in current models. Their inability to simulate realistic responses after sustained significant defocus blur is a serious problem. It means that our best current models of accommodative control cannot handle everyday clinical situations correctly, such as uncorrected refractive error, functional presbyopia, and fogging lenses.

Although the focus of this article is on accommodation alone, it is worth noting that this issue also presents some difficulty for current models of accommodative convergence. Empirically, when a normal observer views a stimulus with one eye, a change in stimulus distance from far to near elicits not only an accommodative response, but also a nasalward movement of the occluded eye, so as to increase the convergence in a way that would help to null the retinal disparity if viewing were binocular. This accommodative convergence is taken to reflect a neural crosslink signal between the accommodative and vergence control systems.

**Figure 3.**
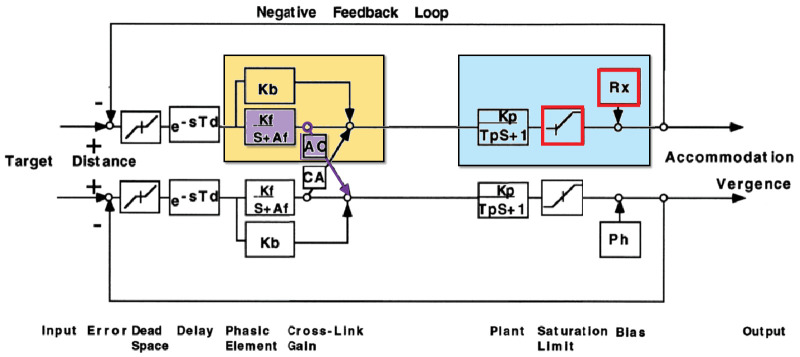
Reproduced from Figure 1b of Schor (1999), showing a model for the control of vergence and accommodation. To facilitate comparison with the present models, we have highlighted the accommodative control system in gold and the ocular plant in blue, as we do in diagrams of the present models. Note that since this model is nonpredictive, the accommodative control system does not contain a virtual plant. We show this model to highlight how it handles the finite range of accommodation via a saturation block (red), the addition of refractive error (also red), and the accommodative-convergence AC signal, taken to be the output of the integrator (purple).

Many current models, for example, that of Schor (1999) shown in Figure 3, postulate that the accommodative–convergence (AC) signal (shown purple in Figure 3) is simply the output of the leaky integrator (purple) that controls accommodation. This strategy works well for stimuli within the accommodative range. For example, if a functional presbyope is viewing a distant stimulus with one eye occluded, and an experimenter inserts a −1 D lens, the accommodative integrator will increase its output by nearly 1 D and both accommodation and vergence will increase accordingly.

However, now suppose the same experiment is conducted closer than the observer's near point, say with the stimulus at 4 D. Accommodation will be unable to increase in response to the divergent lens, so the sustained defocus will charge the accommodative integrator to very high values. According to current models, therefore, vergence will thus increase by around eight times more than it did previously for the same change in demand. Again, we simulate this example in detail in Figure 10, but the essential point is simple enough to be conveyed here. That is, current models predict a very large increase in the ratio of the accommodative convergence response/accommodation (AC/A) stimulus, that is, stimulus AC/A ratio, for stimuli closer than the near point. There is some qualitative empirical support for such an effect (Alpern, Kincaid, & Lubeck, 1959), but not of the predicted magnitude. Greater increases in stimulus AC/A ratio are, however, seen with partial and complete cycloplegia. In partial cycloplegia, the accommodative amplitude gradually decreases, and the AC/A ratio gradually increases as the depth of cycloplegia increases (Christoferson & Ogle, 1956). In complete cycloplegia, the amplitude of accommodation reaches zero and the AC/A becomes nearly infinite (Morgan, 1954).

### The solution: Control based on correctable defocus

**Figure 4.**
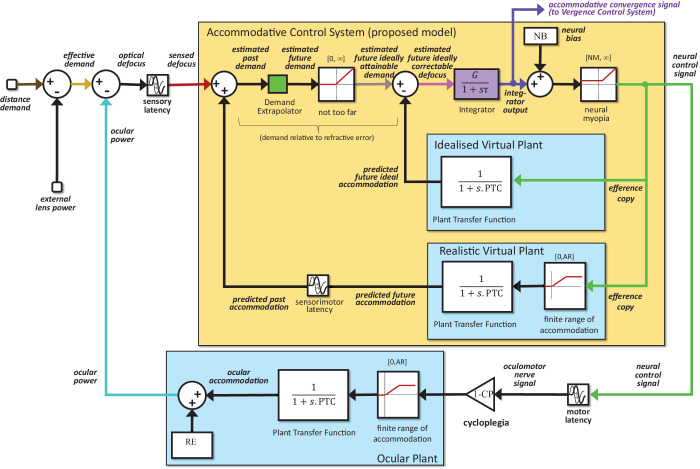
Diagram of the full proposed model. The colors used for the distance demand, effective demand, ocular power, sensed defocus, estimated future attainable demand, estimated future correctable defocus, integrator output, and neural control signal match the colors used to plot these signals in the results figures. The purple arrow at the top shows where the accommodative convergence (AC) signal is drawn off and fed into the vergence control system (not modeled in this article). Refractive error enters after the physical plant in the outer feedback loop and external lenses are subtracted from the physical demand at the input. In subsequent model figures (Figures 5–7), this external feedback loop is not shown; we show only the golden accommodative control system block, whose input is the sensed defocus and whose output is the neural control signal.

In this article, we present an anti–wind-up adjustment to previously published models that addresses all these problems. Our model can produce more realistic simulations of accommodation in the presence of refractive error, fogging lenses, and functional presbyopia, while keeping the output of the accommodative integrator more consistent with the empirical constraints on the AC signal. We also model the relaxation of accommodation seen with pharmacological cycloplegia, where the ciliary muscle is relaxed more completely than is otherwise possible, so that the plant assumes the minimum possible optical power. To capture this effect, we postulate a form of neural myopia: a lower bound on the neural signal sent to the ciliary muscle. For ease of reference, our final proposed model is shown here in Figure 4, and we briefly explain its key features here (see Methods for a detailed description).

Our key idea is that the blur-related signal driving accommodation is not a defocus, but a correctable defocus (pink arrow in gold box in Figure 4). Current predictive models already propose that the brain reconstructs an estimate of stimulus demand from defocus, using a realistic virtual plant. We now propose that this estimated demand is clipped to values that are considered attainable, that is, not beyond the far point, before an estimate of future accommodation is subtracted so as to recover correctable defocus. It is this correctable defocus that is used as the control signal for accommodation. This process ensures that the integrator is not wound up by uncorrectable defocus owing to unattainably distant demands.

We further propose that, in this computation of attainable demand and correctable defocus, the brain uses an idealized virtual plant that takes account of the fact that accommodation cannot be negative (i.e., the ocular lens cannot be divergent), but does not take account of the finite accommodative range. This inaccuracy turns out to be helpful, because it allows stimuli closer than the near point to produce accommodative vergence.

In the Methods, we go through all components of the model and explain how it works in detail. In the Results, we show simulations side-by-side for the original model and for our proposed new model, demonstrating both the problems with the original model and how our new model fixes them.

## Methods

All the models in this article conform to the general structure shown in Figure 1, reviewed in detail in Read et al. (2022).

The internal structure of the ocular plant block is shown in Figure 2. It receives as input the signal to the ciliary muscle, and gives as output the optical power of the eye. Note that in Read et al. (2022) the output was described as “accommodation,” because we were then not considering refractive error and so ocular power was equal to accommodation.

The basic transfer function of the plant is a leaky integrator, equivalently a first-order low-pass filter, specified by its time constant, the model parameter *PlantTimeConstant* (abbreviated PTC in the figures), which in this model is set to 156 ms (Table 1). The saturation block before the plant transfer function imposes the finite range of accommodation. A saturation block is a nonlinear circuit element which has lower and upper limits [*L,* *U*]. Inputs within this range are passed through unchanged; for inputs less than its lower limit *L* or greater than the upper limit *U*, the output is *L* or *U*, respectively. In this case, the lower limit is 0 D and the upper limit is the model parameter *AccomRange*, abbreviated AR, also in diopters. This value represents the finite range of accommodation that declines with age. Representing this as a saturation block before the plant transfer function is clearly an oversimplification. Really, we should start with a more complex model of the plant, including its different components such as zonular fibers, lens capsule, and so on (cf, Figure 6 of Read et al., 2022) and correctly represent the nonlinearities associated with ageing. However, we chose this as an acceptable starting point. Schor (1999) adopted a similar approach, but placed the saturation block after the plant transfer function. Because the plant gain is 1, the maximum steady-state accommodation is the AR, regardless of whether the saturation block is placed before or after the plant transfer function; however, we have found that placing it before gives a more realistic approach to this limit.

**Table 1.** Parameter values for the Simulink model supplied with the article and used to obtain the results (except where stated otherwise). These values are visible in the Simulink Model Workspace, and can be altered there if desired. Notice that the actual rest focus of the model, that is, the asymptotic ocular power in open-loop mode, is equal to the parameter RestFocus only when RestFocus > (NeuralMyopia + RefractiveError). When the parameter RestFocus is less than this, the model's actual rest focus is equal to (NeuralMyopia + RefractiveError).

Parameter	Name in simulink workspace	Abbreviation in figures	Value
Sensory latency	*SensoryLatency*		0.20 s
Motor latency	*MotorLatency*		0.10 s
Time constant of plant	*PlantTimeConstant*	PTC	0.156 s
Gain of integrator	*FastGain*	G	8.0
Time constant of integrator	*FastTimeConstant*	t	2.5 s
Neural bias (constant value added onto the integrator output)	*NeuralBias*	NB	1.4 D
Neural myopia (minimum value of the neural control signal for accommodation)	*NeuralMyopia*	NM	0.2 D
Optical refractive error (as measured under cycloplegia)	*RefractiveError*	RE	0 D *or as stated*
Accommodative range	*AccomRange*	AR	4 D *or as stated*
Cycloplegia	*Cycloplegia*	CP	0 *or as stated*

Finally, we add on *RefractiveError*, the observer's refractive error, also in diopters. Note that, in this article, we are considering the refractive error of the eye, rather than the power of the lens used to correct it. Ocular refractive errors are, therefore, positive for myopes (a myopic eye has too much optical power for its length and needs correction with negative lenses) and negative for hyperopes.

As an example, consider an observer with an AR of 4 D, typical for someone in their late forties. An emmetropic observer with this range can focus on stimuli from 0 D to 4 D (infinity down to 25 cm). A myope with +2 D of refractive error can focus on stimuli from 2 D to 6 D (50 cm to 17 cm), whereas a −2 D hyperope can focus on stimuli from −2 D to 2 D (i.e., can focus unaided on stimuli from infinity down to 50cm, and can also tolerate plus lenses of up to +2 D without blur). All model parameters are given for reference in Table 1.

### Simplified model of accommodative control used in this article

For simplicity, in this article we use a stripped-down version of the model developed in Read et al. (2022), removing elements that are irrelevant to the point at issue. To this end, we remove noise, the clipped proportional signal, and the slow integrator, as well as continuing to neglect the pulse component of the response to step changes. We retain a bias signal controlling rest focus. (If we retained the slow integrator, the latencies would potentially be even longer, since both the slow and the fast integrator would charge up.)

**Figure 5.**
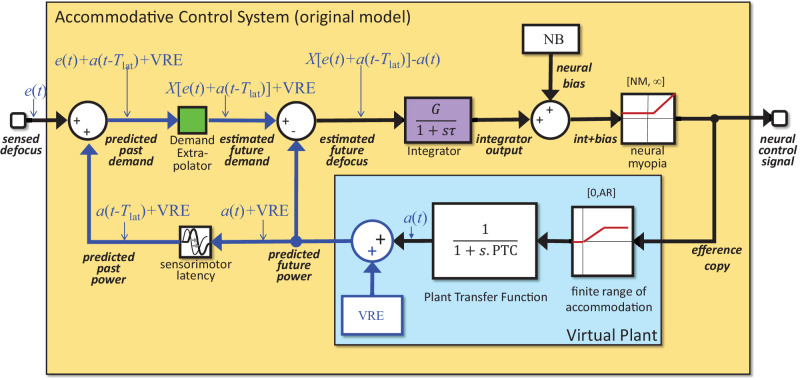
Structure of the accommodative control system (yellow block in Figure 1) in the version we refer to as the “original model.” This is as in Read et al. (2022), but with refractive error and a minimum neural accommodative signal added and with extraneous elements (noise, slow integrator, proportional signal) not relevant to this article removed for simplicity. Signals drawn in blue are those affected by the VRE inside the blue virtual plant block. The virtual plant is identical to the Ocular Plant block shown in Figure 2. The output of the green Demand Extrapolator block is shown as a general function *X*, which is assumed to be linear so that *X*(input + constant) = *X*(input) + constant. In our Simulink models, *X* is the identity, that is, the demand extrapolator simply assumes that the stimulus demand will remain at its current value. Here and subsequently, we use the adjective “predicted” (e.g., predicted future power) to refer to the control system's computation of external signals, which are error free in our model (because they depend only on the system's own behavior and on the properties of the ocular plant, which could in principle be learned by the brain and are known to our model). We use the term “estimated” (e.g., estimated future demand) to refer to the control system's computation of external signals that are not fully within its control, for example, because they depend on the self-motion of scene objects. NB and NM are abbreviations for the model parameters NeuralBias and NeuralMyopia, respectively. VRE, virtual refractive error. As discussed in the text, where demand extrapolation is linear, the VRE can be set to zero without loss of generality. Notice that, here and subsequently, to limit clutter we do not show the accommodative convergence signal, but it remains equal to the integrator output.

Figure 5 shows the contents of the accommodative control system block in Figure 1, as it would be in this stripped-down version of the original model. This figure contains a virtual internal model of the accommodative plant, shaded light blue in the figures, which is used to generate internal predictions about future ocular power, demand and defocus. See our earlier article for an in-depth discussion of this predictive control. In brief, the input signal to the accommodative control system is the sensed defocus, assumed to be computed from retinal information, such as blur and higher order aberrations, longitudinal chromatic, and aberrations. However, to avoid control problems such as overshoot and ringing, the neural control signal is not generated directly from this sensed current defocus, but from the brain's estimate of the likely future defocus, taking into account the predicted changes in accommodation based on the control signal already sent to the eye. These are computed via an efference copy of the neural control signal, which is fed into a virtual plant model, outlined in light blue. The output of the virtual plant is the eye's predicted future optical power. Figure 5 shows how this factor is delayed and then added onto the sensed defocus to compute the predicted past demand. The green block labeled demand extrapolator” in Figure 5 uses this to estimate the future demand. (This block was referred to as the demand predictor in our previous article. We now feel the term demand extrapolator is more helpful, because it emphasizes that stimulus demand is not necessarily within the control of the observer and, thus, cannot in principle always be predicted perfectly.) In our Simulink models, the demand extrapolator in fact simply feeds through its input, that is, it assumes the effective demand will remain constant for at least a time equal to the sensorimotor latency. The predicted future power is then subtracted from this estimated future demand to result in the estimated future defocus, which is the key signal used for accommodative control.

The estimated future defocus is fed into the Integrator block, shaded purple in the figures. This corresponds with the fast integrator in the full model. It is a leaky integrator, which is the heart of the model's control of accommodation. Its gain and time constant are central to the model's behavior. The model also includes a neural bias signal, which is added onto the integrator output. As discussed elsewhere in this article, this bias helps to set the rest focus adopted in open-loop viewing.

In the Results section, we demonstrate that the original accommodative control system shown in Figure 5 predicts unrealistic latencies owing to integrator wind-up, and we will discuss various alternatives. Before proceeding to this discussion, it will be helpful to discuss various aspects that apply to all models discussed in this article.

### Modeling rest focus as a neural bias

When we run the model in open-loop mode with the defocus signal clamped at zero, as if viewing through pinholes, the estimated future defocus and thus the integrator output must also be zero. If accommodation were driven solely by the integrator output, the neural control signal should, therefore, take on its minimum value, and the ocular power should be close to the closed-loop value for distant stimuli. Yet this is not observed empirically. Instead, accommodation is substantially higher: there is a nonzero “rest focus,” on average around 1.4 D, even after correction for refractive error (Fisher, Ciuffreda, & Levine, 1987; McBrien & Millodot, 1987; Rosenfield, Ciuffreda, Hung, & Gilmartin, 1993). This finding implies a neural bias signal that operates even in the absence of any input to the system. To account for this factor, our model includes a constant signal added on to the integrator output (block labelled “neural bias” at the top of Figures 4^5^,^6^–7). The value of this signal is the parameter *NeuralBias* of our model, abbreviated NB (Table 1). Note that the rest focus itself, and thus the accommodative lag/lead, depends on refractive error as well as on the amount of neural bias; this effect can be seen in Figure 7.

**Figure 6.**
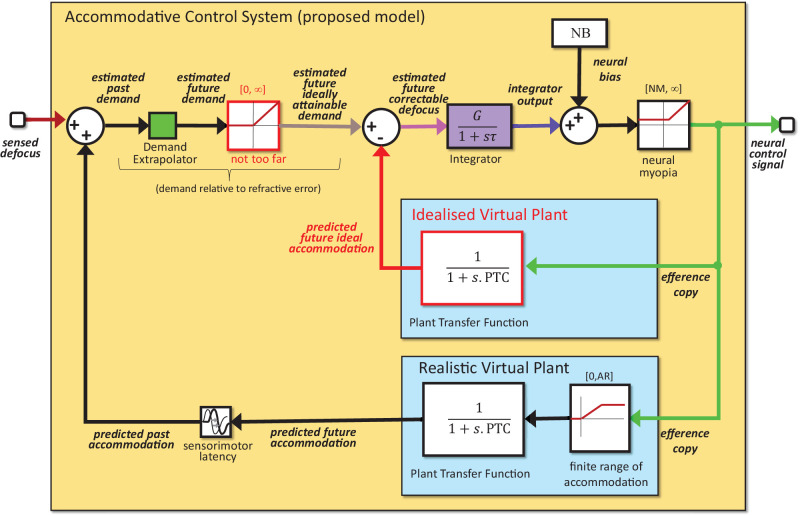
Accommodative control system of the proposed new model. As in Figure 5, but we have removed the refractive error term from the virtual plant, because as shown above this does not change behavior. In consequence, the signals labeled “demand” actually represent “demand minus refractive error” We have added a saturation block, outlined in red and labeled “not too far.” There are now two virtual plants: a “realistic virtual plant” outputting the predicted future accommodation, with the same finite range as the physical plant, and an “idealized virtual plant” outputting the ideal accommodation, with no upper limit. The latter is important for computing an appropriate accommodative convergence signal. Signals shown in color match colors used in results figures.

**Figure 7.**
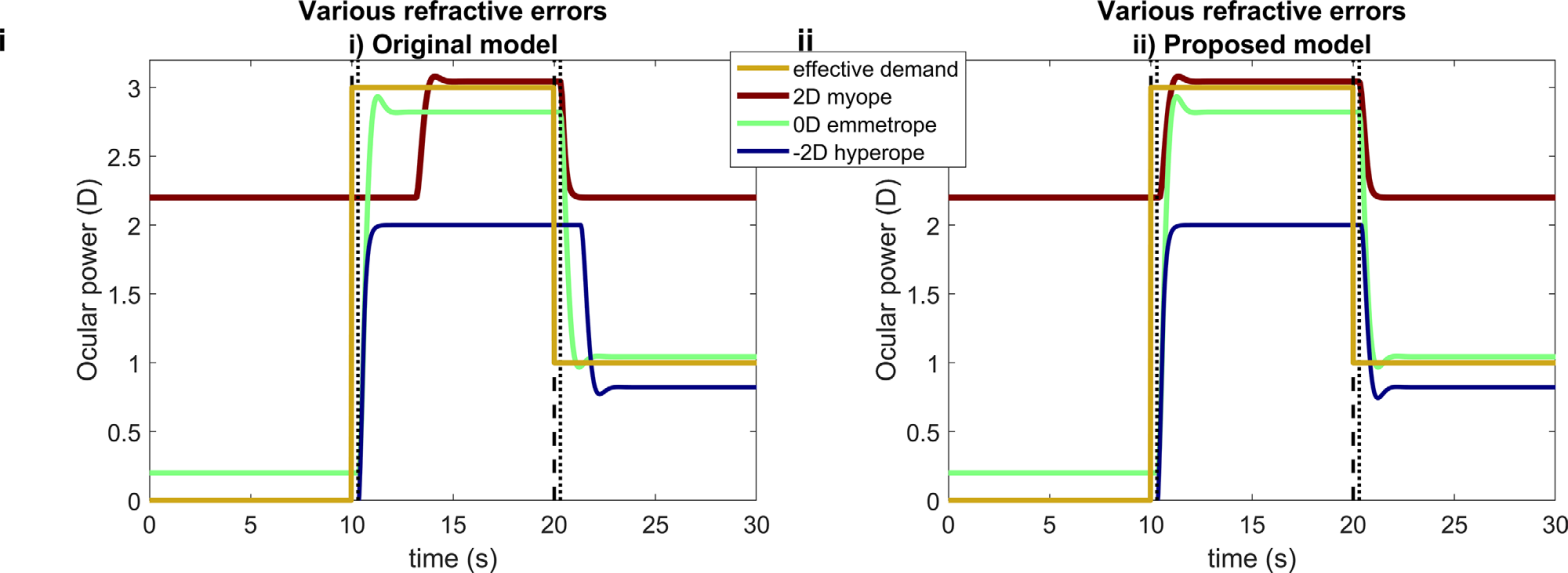
Time courses of ocular power for a stimulus which is initially at infinity (0 D), then moves to 33 cm (3 D) at *t* = 10 seconds, then moves back to infinity at *t* = 20 seconds, for three different functionally presbyopic observers with accommodative range of 4 D and refractive errors indicated in the legend, for **i**) the original model (Figure 5) and **ii**) our proposed new version (Figures 4 and 6). The myope and emmetrope have different steady-state responses to the 3-D stimulus; this is because of different accommodative lag owing to different rest focus (both have the same neural bias, 1.4 D, but rest focus also depends on refractive error). Dashed vertical lines mark the time of step changes in demand, and dotted lines these plus the sensorimotor latency of 300 ms. This figure was obtained with Matlab file PlotOcularPowerDifferentRE.m.

### Modeling the relaxation of accommodation with cycloplegia: Neural myopia

With a positive neural bias, the closed-loop response for stimuli at optical infinity is predicted to be positive instead of exactly zero. If control were purely linear, then by using fogging lenses to apply sufficient negative demand, it would be possible to counter a positive neural bias and fully relax accommodation by purely optical means. Empirically, however, this is not possible in most people: even after relaxing accommodation as much as possible optically, pharmacological cycloplegia causes a further decrease in ocular power (Bagheri et al., 2018; Hiraoka et al., 2013; Yoo, Cho, Kim, & Baek, 2017). This finding suggests that the oculomotor nerve signal has a minimum value below which it cannot decrease, which in turn keeps accommodation at a minimum value: effectively, a form of neural myopia. Cycloplegia blocks this minimum signal, enabling accommodation to relax completely.

To account for this, we added the saturation block labeled “neural myopia” in Figures 4, 5, and 6. This saturation block has no upper limit (i.e., the upper limit is set to infinity), but its lower limit is set to the parameter *NeuralMyopia*, abbreviated NM (Table 1). Inputs exceeding NM are passed through unchanged, while all inputs less than NM result in output NM. Unlike the linear neural bias, this nonlinear effect cannot be overcome with negative demand owing to fogging lenses. Cycloplegia abolishes it via a downstream gain, explaining the additional relaxation observed with cycloplegia.

### Note on the definition of tonic accommodation

“Tonic accommodation” is often used to refer to the neural signal driving the rest focus measured in darkness or in open loop (McBrien & Millodot, 1987; Rosenfield et al., 1993). This factor corresponds with our neural bias parameter, NB. However, other workers define “tonic accommodation” as “the eye's normal functional state for distance” in closed loop (Hiraoka et al., 2014), which would correspond with our neural myopia parameter, NM, plus any refractive error. To avoid confusion, we do not use the term tonic accommodation.

### Note on the definition of refractive error

In this article, we define refractive error as the optical power of the eye when accommodation is fully relaxed. However, in general, accommodation cannot be relaxed fully unless the eye is completely cyclopleged. It follows that our *RefractiveError* parameter represents the optical power of the eye under cycloplegia. In our model, the optical power measured when the uncyclopleged eye views an object at infinity, or through plus lenses, is *RefractiveError+NeuralMyopia*. In some empirical studies, what is referred to as the refractive error may, therefore, correspond in our model with the optical refractive error plus neural myopia.

### The brain does not need to know its own refractive error

In Figure 5, the virtual plant model is shown as including a virtual refractive error (VRE) term. However, the value of this term does not affect the behavior of the model, at least when the demand extrapolator block is linear. To show this, the small blue labels in Figure 5 mark on the values of selected signals, starting with the accommodation output by the virtual plant, *a*(*t*), and the sensed defocus error, *e*(*t*). As shown in Figure 5, the value of VRE affects the predicted ocular power and thus the estimate of demand fed into the Demand Extrapolator block. Let's denote the function performed by the Demand Extrapolator block as X(*input*). If the function *X* is linear, then by definition X(*input* + VRE) = X(*input*) + VRE. The VRE term then cancels out when the predicted future power is subtracted from the estimated future demand. Thus, the estimated future defocus, drawn pink in Figure 5, is independent of the value of VRE. The value of VRE affects the internal signals shown in blue, but has no effect on the overall transfer function of the accommodative control system. VRE can thus, without loss of generality, be set to zero, and we will adopt this simplification in the rest of the article. Thus, none of the models discussed in this article require us to postulate that the brain has any information about the ocular refractive error.

### Developing a new model

As we show in the Results, the problems with current models stem from the same basic problem: excessive integrator output during exposure to stimuli beyond the accommodative range, which then leads to long latencies to subsequent stimuli within range. To fix the issues, we assume that neural control is sophisticated enough to recognize that sustained charging in such a situation is unhelpful, and takes steps to avoid passing sustained uncorrectable defocus to the integrator.

Figure 6 shows the accommodative control system of our proposed new model (see Figure 4 for the complete system, including stimulus and eye). To avoid wind-up, our new model postulates that the estimated future demand passes through an additional saturation block, outlined in red in Figure 6 and labeled “not too far.” If the estimated future demand is beyond the observer's far point, its output is zero. This means that stimuli whose accommodative demand is unattainable because they are beyond the observer's far point are treated as if they were at the far point. However, the saturation block has no upper bound, so stimuli that are closer than the near point are treated normally, as if these demands were attainable, as indeed they could be by an ideal young eye. The output of this saturation block is, therefore, labelled the “estimated future ideally attainable demand.”

The “not too far” saturation block stops the integrator being wound up by uncorrectable negative defocus, caused by unattainable “too far” demand. We now need to ensure that the integrator is not wound up by uncorrectable positive defocus, caused by an unattainable too near demand. To achieve this, we postulate that, when estimating future correctable defocus, the brain subtracts off the accommodation predicted for an ideal eye with unlimited range of accommodation, rather than for its own limited range. This is shown by the block labelled “idealized virtual plant” in Figure 6. This avoids integrator wind-up because the predicted future ideal accommodation matches the estimated future ideally attainable demand, resulting in very low steady-state input to the integrator. The fact that both are unattainable in practice makes no difference to the steady-state response.

However, there is one important difference in the time course. Because the idealized virtual plant still has the same finite time constant as the real plant, an increase in the estimated ideally attainable demand takes time to produce an increase in the predicted future ideal accommodation. There is thus a transient correctable defocus signal which produces a change in integrator output. We propose that this transient signal is what produces the AC response observed for increases in too near demand.

Conversely, decreases in too far demand are clipped at the saturation block and never feed through to produce even a transient change in the defocus signal. This explains why such decreases do not produce an accommodative–divergence response. This is how our proposed accommodative control system, Figure 6, accounts for the asymmetry between the accommodative–vergence responses for stimuli that are too near versus too far.

### Simulation details and model parameters

Simulations were run in Matlab Simulink, R2019a, using a fixed-step solver with timestep set to 1 ms. All models and code are provided with the article, and are available to download at https://doi.org/10.25405/data.ncl.21909279.

## Results

### Current models produce unrealistically long latencies after stimulation beyond the accommodative range

The central topic of this article is the unrealistically long latencies observed in current models after exposure to demands beyond the accommodative range of the simulated observer. This is demonstrated in Figure 7. Here and in subsequent figures, results for the original model are shown on the left and results for our proposed new model are shown on the right, to facilitate comparison.

In Figure 7, the time course of the stimulus effective demand is shown in yellow. It steps from 0 D at time zero up to +3 D at *t* = 10 seconds and back down to 1 D at *t* = 20 seconds. The accommodative response is shown for three simulated observers with different amounts of refractive error: an emmetrope with 0 D, a myope with +2 D, and a hyperope with −2 D. All simulated observers are assumed to have the same total accommodative range, 4 D, and the same minimum neural accommodative signal, 0.2 D.

The emmetrope and hyperope accommodate close to 0 D for the 0-D stimulus, but the myope only gets to around +2.2 D, reflecting their refractive error plus the minimum neural accommodative signal. They thus experience defocus while viewing this distant stimulus. When the stimulus demand steps up to 3 D at *t* = 10 seconds, the emmetrope and myope both successfully get close to 3 D, but for the −2-D hyperope, +3 D is out of range. Finally, when the stimulus demand steps down to 1 D at *t* = 20 seconds, the emmetrope and hyperope successfully accommodate close to this value, but the +2-D myope cannot focus on objects this far away.

These steady-state values are as expected for the specified refractive errors, accommodative range, gain and minimum neural accommodative signal. However, notice the latencies for the original model (Figure 7i). At *t* = 10 s, the emmetrope and hyperope begin responding immediately after the expected sensorimotor latency of 300 ms. However, the myope does not begin responding until some seconds after the step change in demand. Similarly when the demand drops to 1 D at *t* = 20 seconds, and the other simulated observers begin relaxing accommodation after 300 ms as expected, the hyperope does not begin responding until 1,700 ms after the stimulus change. As discussed, these long latencies are extremely unrealistic and, thus, indicate a problem with current models. Figure 7ii confirms that our proposed new model does not show these long latencies.

#### The excessive latencies are caused by excessive integrator output in current models

**Figure 8.**
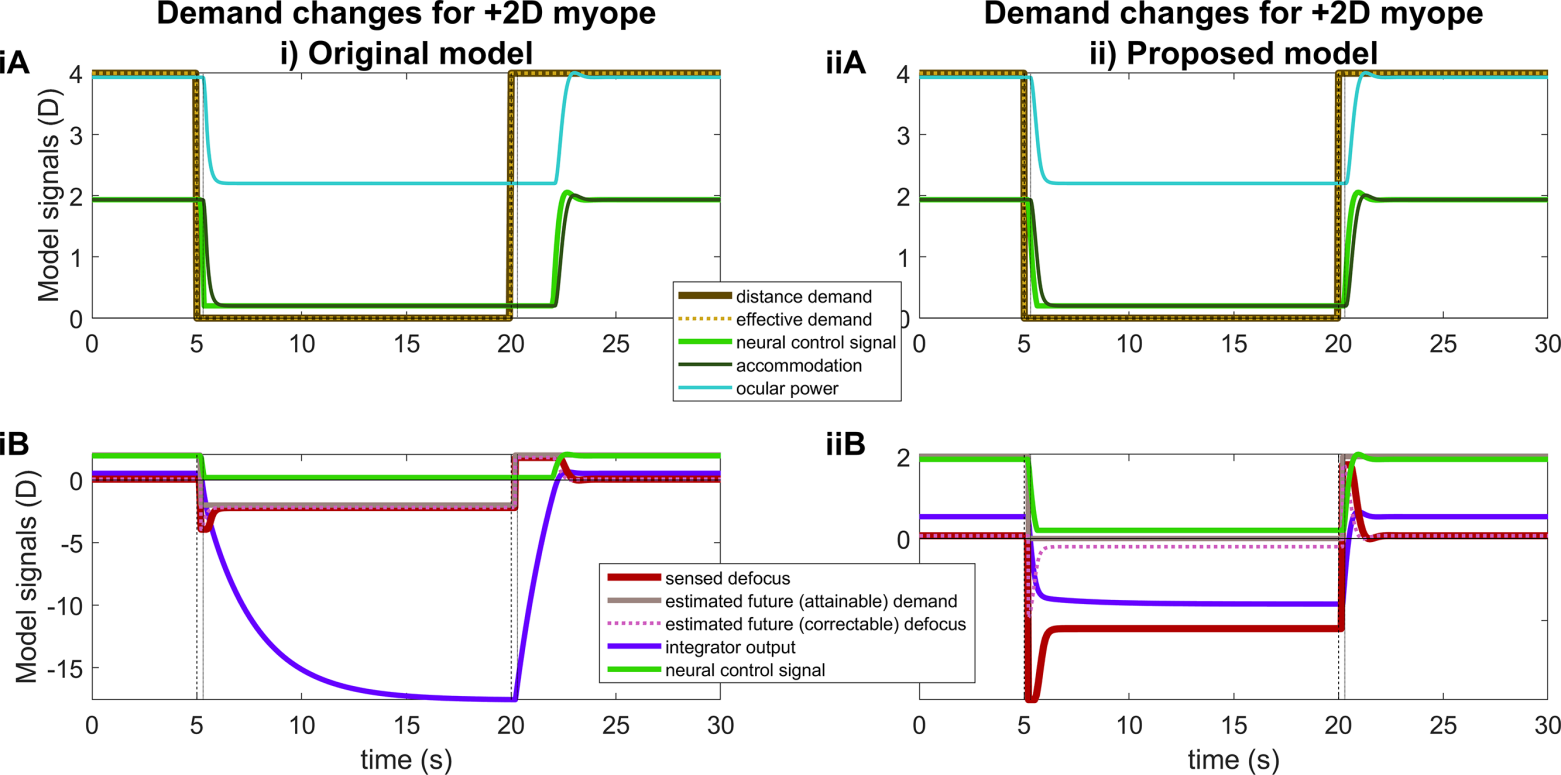
Time -courses of signals for a model myope with +2-D refractive error and accommodative range of 10 D, when stimulus demand steps from 4 D to 0 D at *t* = 5 seconds and then back from 0 D to 4 D at *t* = 20 seconds (vertical dashed lines). In this example, the effective demand is equal to the distance demand; we show both for consistency with subsequent figures. The vertical dotted lines mark 300 ms after each stimulus change, that is, the usual sensorimotor latency. In the original model, (**i**), the initial relaxation of accommodation occurs with this latency after *t* = 5 seconds, but the subsequent accommodative response is delayed over 2 seconds beyond the usual latency, because of internal adaptation to the previous tonic defocus error, as shown in (**B**). See Figures 5 and 6, respectively, for where the internal signals are taken from in the two models; the same colors are used here as in Figure 4. In the figure legend, “(attainable)” and “(correctable)” apply only to the proposed model (**ii**) (see Figure 6), because the original model (**i**) (see Figure 5) does not make these distinctions. Recall that in both models, estimated demand is relative to the refractive error. Note the very different *y* axes in (**iB**) versus (**iiB**). This figure was obtained with Matlab file PlotSignalsForMyope.m.

Figure 8iA illustrates the underlying reason for the long latencies in the original model, for the example of an uncorrected +2-D myope observing a stimulus which is initially at a distance of 4 D, then at *t* = 5 seconds switches to 0 D, then at *t* = 20 seconds switches back to 4 D. This demand time course is shown in Figure 8iA by the thick brown line with yellow dots. The remaining curves show various signals for the myopic observer. The cyan line shows the time course of the ocular power, and the dark green line shows the time course of accommodation, which is 2 D lower than the ocular power owing to the myopic refractive error. When the stimulus is at 4 D, the ocular power is close to 4 D (a little less owing to accommodative lag), so the stimulus is in focus. When the stimulus moves to 0 D at *t* = 5 seconds, the myope relaxes accommodation to the minimum permitted by neural myopia, NM = 0.2 D, but owing to their +2-D refractive error they cannot bring the ocular power below +2.2 D. They. therefore. experience a steady-state defocus error of −2.2 D (red and pink traces in Figure 8iB).

This negative defocus error feeds into the integrator (purple trace in Figure 8iB). The output of the integrator thus becomes more and more negative, eventually asymptoting to a value around −17 D, representing its gain of 8 multiplied by the constant defocus error of −2.2 D. The desired command is equal to the output of the integrator plus the neural bias, here 1.4 D. However, the actual motor signal cannot be negative, so remains fixed at 0 (light green trace in Figure 8iAB). The lens remains fully relaxed, but the refractive error means that the system continues to experience −2 D of defocus.

At time *t* = 20 seconds, the stimulus changes back to +4 D and after the sensory latency of 200 ms, the sensed defocus thus jumps to +1.8 D. The input to the integrator thus becomes positive, and the output of the integrator rises rapidly. However, because the neural control signal is not allowed to be negative, no change in motor signal occurs until the combination of the integrator output and the neural bias exceeds the neural myopia. In this model the neural bias is 1.4 D and neural myopia is 0.2 D (Table 1), so this occurs once the integrator output exceeds −1.2 D. From the integrator's starting point of −17 D, this takes around 2 seconds to occur. Thus, the motor signal does not become positive until 2 seconds after the increase in demand, and so no change in ocular power or sensed defocus is seen until that point. The original model thus predicts that, after far viewing, the myope has an enormous, 2-second latency before they begin refocusing on near objects.

In contrast, in the proposed new model (Figure 8ii), the lower bound of the red “too far” saturation block shown in Figure 6 means that the estimated future attainable demand is clipped at 0 D, instead of being −2 D, as it was in the original model (Figure 8iB; recall that estimated demands within the accommodative control system are expressed relative to the refractive error). Accordingly, although the sensed defocus is again −2.2 D (red trace in Figure 8iiB), the estimated future correctable defocus is now only −0.2 D (dashed pink trace in Figure 8iiB: 0 D attainable demand minus 0.2 D accommodation). The integrator thus asymptotes to only −1. 6D (−0.2 D times its gain of 8; purple trace in Figure 8iiB).

When the stimulus then jumps 2 D to +4 D at *t* = 20 seconds, the defocus error also jumps 2 D, from −2.2 D to +1.8 D. The estimated future demand relative to refractive error is now +2 D, which is within the range of the myopic observer, and so the estimated future attainable demand also becomes +2 D. The estimated future correctable defocus therefore transiently becomes the actual defocus, +1.8 D. Because the integrator starts from a value of only −1.6 D, it takes virtually no time to reach the threshold value of −1.2 D, beyond which the motor signal starts to increase. Accommodation therefore starts increasing virtually as soon as the sensorimotor latency allows, that is, 300 ms after the stimulus change (dark green trace in Figure 8iiA). As accommodation increases, defocus falls and the system enters a new steady state.

#### External lenses

**Figure 9.**
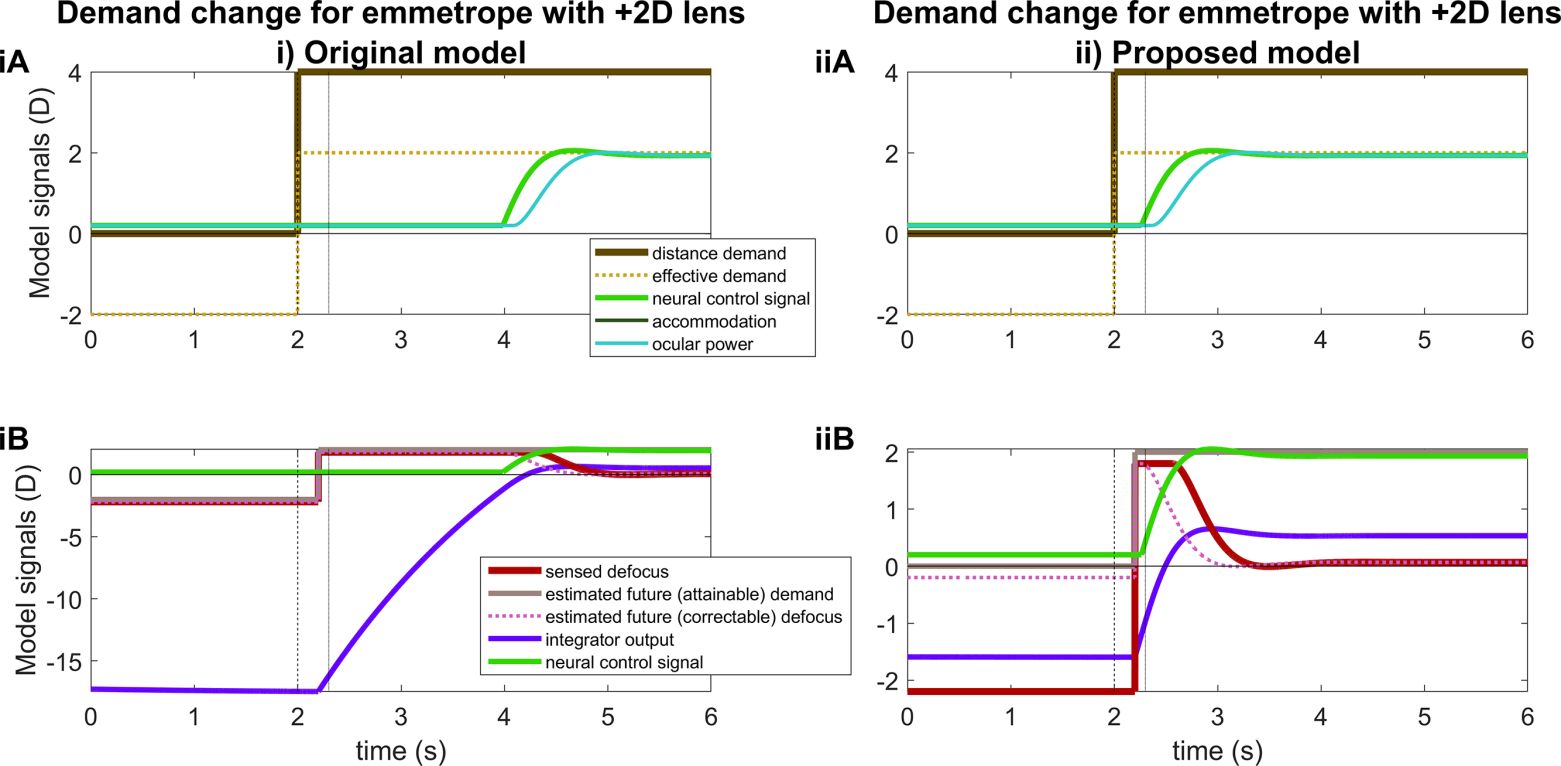
Time courses of signals for a model emmetrope viewing a stimulus at infinity through a +2-D external fogging lens, before the distance demand steps from 0 D to +4 D at *t* = 2 seconds (vertical dashed line). The original model (**i**) predicts a long delay before the emmetrope focuses on new effective demand at 2 D, because of adaptation during the preceding period of fogging. Dotted lines mark the time a response would normally begin, that is, the sensorimotor latency (here 300 ms) after the change in demand. Note the different scales of the *y* axes in (**iB**) versus (**iiB**). Other details as for Figure 8. This figure was obtained with Matlab file PlotSignalsForEmmetropePlusLens.m.

Similar excessive predicted latencies are also seen when refractive error is simulated with external lenses. For example, Figure 9 shows the model predictions for an emmetrope viewing through a +2-D fogging lens. Initially, the observer is viewing a stimulus physically at infinity, so the effective demand is −2 D (yellow dotted line in Figure 9A). They experience a steady −2.2 D of defocus blur (red, dotted pink lines in Figure 9B) owing to the +2 D plus lens and their inability to relax accommodation below their neural myopia of 0.2 D. In the original model, this negative defocus error feeds into the integrator, which therefore asymptotes at a large negative value (again around −17, representing its gain of 8 multiplied by the constant defocus error of −2.2 D). Subsequently, when the stimulus moves to a distance of +4 D, the emmetrope accommodates to +2 D, so that the stimulus seen through the +2 D lens is in focus. However, owing to the previous steady-state defocus and the resulting large negative value of the integrator at the time of the change, there is a delay of >2 seconds before they make this response and, thus, before sensed defocus decreases.

In the proposed new model, there is again a large sensed defocus error of −2.2 D, but the estimated attainable demand is again 0 D. This factor ensures that the estimated future correctable defocus remains at −0.2 D (pink dotted line in Figure 9iiB). Thus, the asymptotic output of the integrator is again −1.6 D, despite the sustained large defocus. Thus, when the stimulus moves closer at *t* = 2 seconds, we see ocular power increasing in response just after the 300 ms sensorimotor latency (dashed line at *t* = 2.3 seconds). We do not see the unrealistic 2-second latency shown by the original model in Figure 9iA.

**Figure 10.**
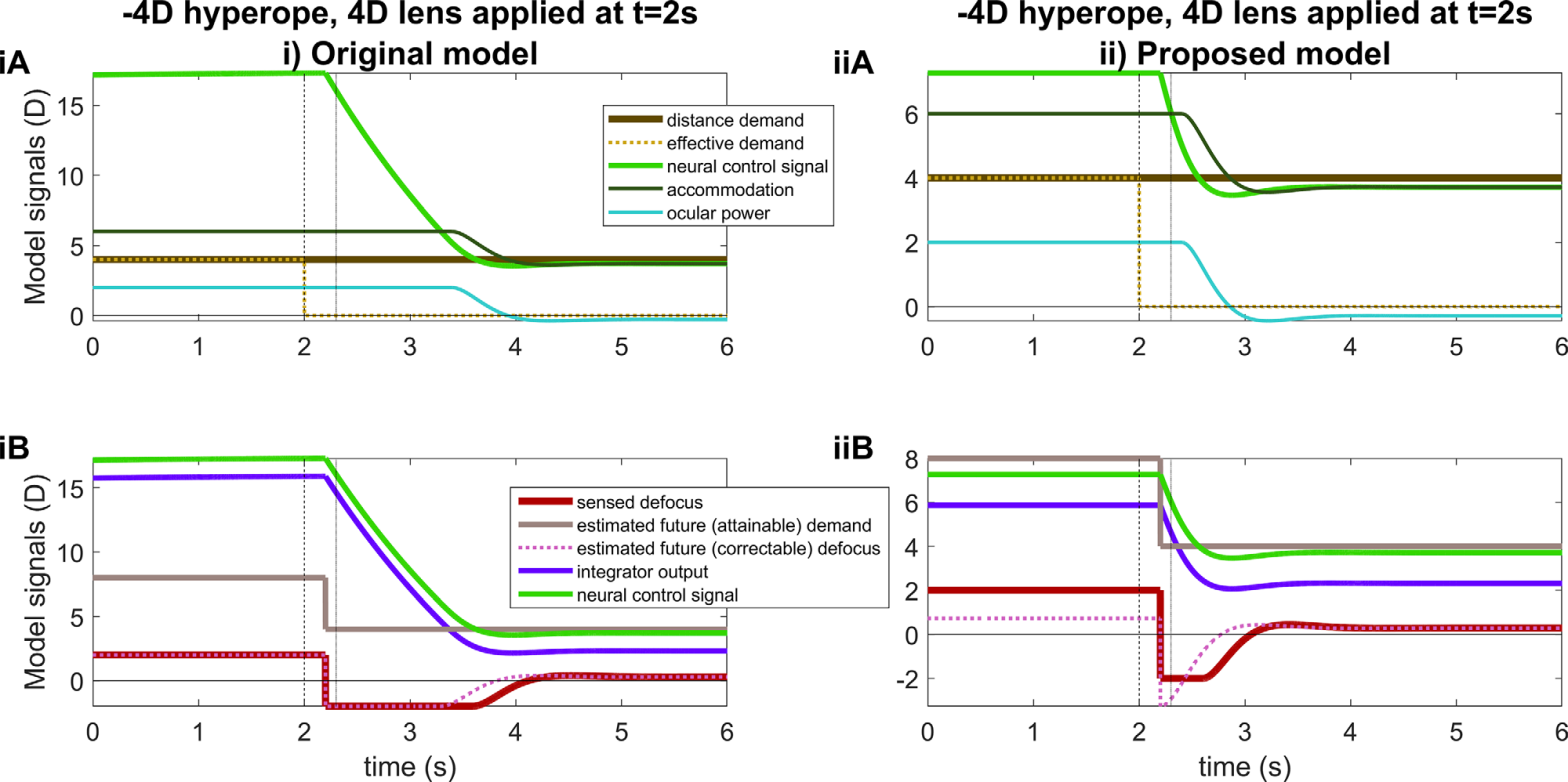
Time courses of signals for a hyperope with a refractive error of −4 D and accommodative range of 6 D, before and after a corrective lens is applied at *t* = 2 s. The stimulus distance demand is constant at +4 D; a corrective lens of +4 D is added at *t* = 5 seconds, reducing the effective demand to 0 D. Other details as for Figure 8. This figure was obtained with Matlab file PlotSignalsForHyperopePlusLens.m.

Figure 10 shows another example. This is for a model hyperope with a refractive error of −4 D and accommodative range of 6 D, who can therefore focus on stimuli from −4 D to 2 D. In this example, the stimulus distance is 4 D throughout (heavy brown line in Figure 10A), closer than their maximum ocular power of 2 D. Thus, when viewed without corrective lenses (*t* < 2 seconds), the demand appears with +2 D of defocus blur even though the hyperope is accommodating as much as they can (accommodation 6 D, ocular power 2 D). At *t* = 2 seconds, a corrective lens of +4 D is applied, fully correcting the hyperopia. The hyperope can now relax accommodation to 4 D and focus on the stimulus. However, because the integrator is charged to 16 D after the prolonged defocus (2 D defocus times gain of 8, purple trace in Figure 10iB), the original model predicts that no relaxation of accommodation occurs until more than 2 seconds after the lens is applied (dark green accommodation trace in Figure 10iA). Similarly the sensed defocus jumps from +2 D to −2 D when the +4 D lens is applied, but no reduction in the magnitude of defocus occurs until ∼1.5 seconds later (red trace in Figure 10iA).

Again, the new model fixes these problems because the integrator was not allowed to charge up to high values. The sensed defocus was still 2 D, reflecting an estimated demand of +8 D relative to the refractive error, but the brain predicted that the idealized virtual plant would be able to accommodate to 7.3 D. It, thus, estimates the correctable defocus as only 0.7 D, so the integrator asymptotes at 5.9 D. Accordingly, accommodation relaxes shortly after the plus lens is applied, just a little after the 300-ms sensorimotor latency (dark green accommodation trace in Figure 10iiA, latency marked with dashed vertical line at 2.3 seconds).

#### AC/A ratio

**Figure 11.**
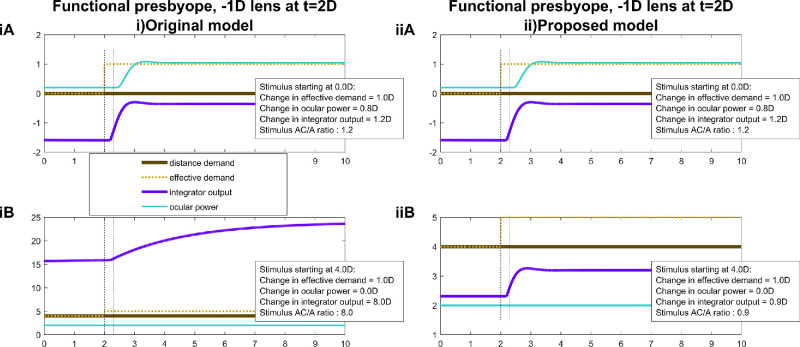
Time courses of signals for a functional presbyope with a maximum ocular power of 2 D, before and after a −1-D diverging lens is applied at *t* = 2 s, as in an experiment to measure AC/A ratio. The stimulus demand is constant, at (**A**) infinity, 0 D and (**B**) the observer's maximum ocular power, 2 D. The “stimulus AC/A ratio” is obtained by dividing the total change in integrator output by the change in the effective demand. This assumes that the accommodative-convergence signal is the output of the accommodative integrator, as in Figure 1, with unit gain. This figure was obtained with Matlab file PlotACAratio.m.

Figure 11 illustrates the problem with the accommodative convergence signal, alluded to in the Introduction. It represents the model's predictions for an experiment designed to measure the AC/A ratio: the observer fixates monocularly, then the experimenter inserts a minus lens and observes the increase in ocular vergence angle owing to the increase in accommodative demand. The stimulus AC/A ratio is the ratio of this change in convergence to the change in stimulus accommodative demand (Alpern et al., 1959; Evans & Pickwell, 2007; Stidwill & Fletcher, 2011). As discussed in the Introduction, in current models the accommodative convergence signal is drawn from the output of the accommodative integrator, multiplied by the AC gain and added onto the output of the vergence integrator (Figure 1). The change in vergence in this experiment is, therefore, proportional to the change in output of the accommodative integrator produced by the addition of the minus lens.

Figure 11iA shows the predictions of the original model when a functional presbyope with maximum ocular power of +2 D views a stimulus at 0 D, with a −1-D lens inserted at *t* = 2 seconds. This all works as expected. When the −1-D lens is inserted, the ocular power increases by approximately 1 D to compensate, reflecting a similar increase in the integrator output. If the change in convergence reflects the change in integrator output, as in Figure 1, then the stimulus AC/A ratio would be proportional to the change in integrator output. In the figure legend, we have recorded this as “stimulus AC/A ratio,” assuming for simplicity that the constant of proportionality is 1. (This stimulus AC/A ratio ends up being a little over 1 (1.2) owing to the neural myopia; see discussion around Figure 14.)

However, Figure 11iB shows the problem encountered if the baseline stimulus is closer than the observer's maximum ocular power of 2 D: at 4 D, in this example. The observer experiences sustained defocus of +2 D, that is, the difference between the demand of 4 D and their maximum accommodation of 2 D. This defocus drives the integrator output to around 16 D (2 D times its gain of 8). Adding the −1-D lens increases the defocus to +3 D, driving the integrator output up to 24 D. This, of course, has no effect on ocular power, but would produce a further dramatic increase in convergence. Whatever an observer's AC/A ratio is when the baseline stimulus is within their accommodative range, the current model predicts that it should be much greater for baseline stimuli closer than the near point: multiplied by the gain of the accommodative integrator.

Figure 11ii shows results for the proposed model. Now, the predicted stimulus AC/A ratio actually decreases slightly when the baseline stimulus is closer than the near point.

**Figure 12.**
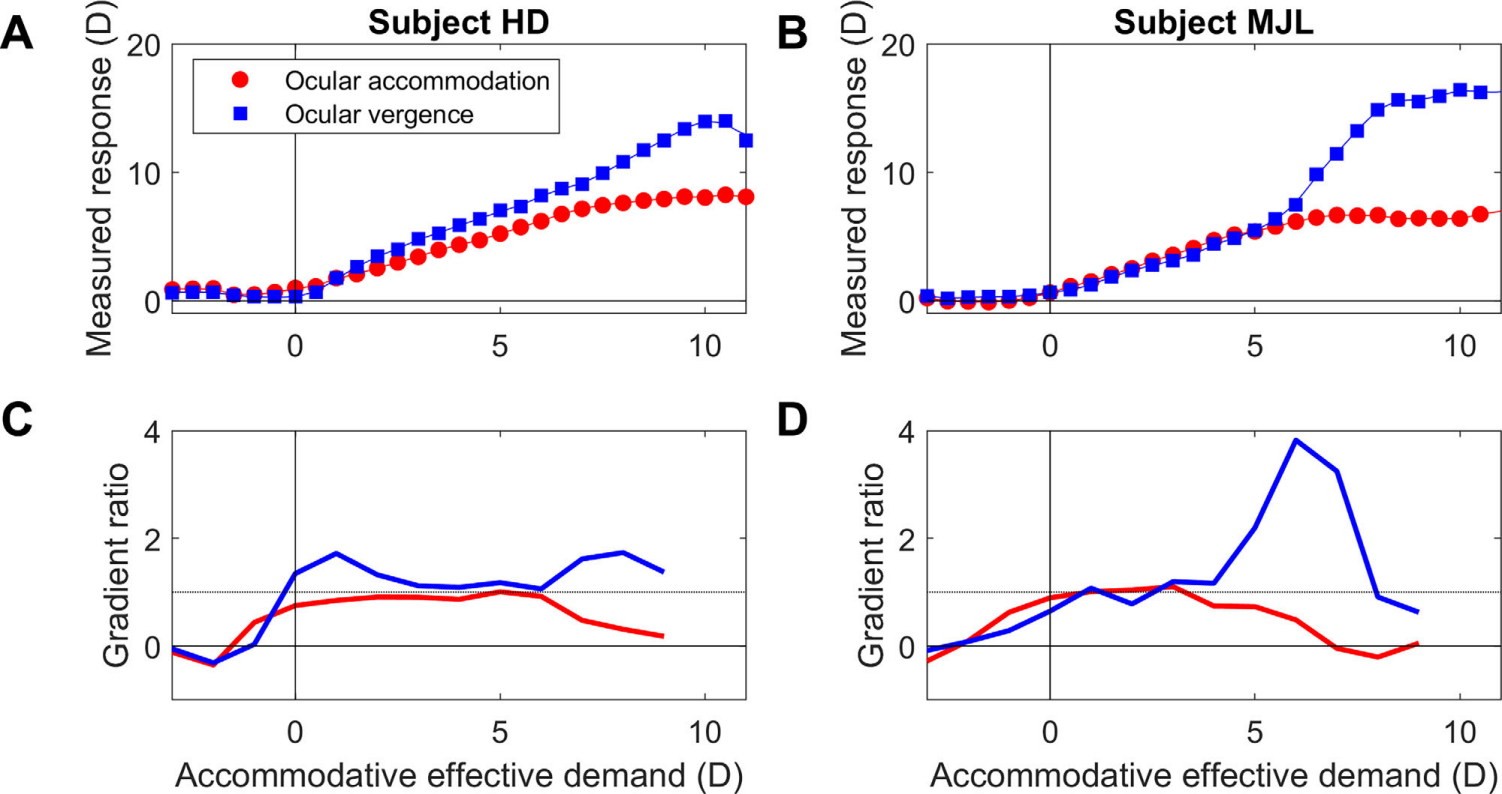
Experimental data digitized from Figure 3 of Alpern et al. (1959) for subjects HD (AC) and MJL (BD). (**A** and **B**) Red disks: accommodative response function, that is, measured ocular accommodation as a function of the effective demand. Blue squares: measured ocular vergence, converted into diopters to facilitate comparison, assuming an interocular distance of 7 cm. This reflects accommodative convergence since the stimulus was monocular. Lines show smoothing splines fitted to data. (**C** and **D**) Red lines: the change in ocular accommodation estimated when accommodative effective demand increases by 1 D from the value shown on the *x* axis, computed using the fitted splines, that is, an approximation to the derivative of the accommodative response function, designed to be comparable to the stimulus AC/A ratio as computed by the gradient method using a −1-D lens. Blue lines: ditto for vergence, that is, the stimulus AC/A ratio. This figure was obtained with Matlab file Fig_AlpernKincaidLubeck1959.m.

The literature does show evidence for a transient increase in AC/A ratio around the near point, but not for all points closer than that. Figure 12 shows data for two subjects, replotted from Alpern et al. (1959). The red disks in Figure 12AB show the ocular accommodation measured during monocular viewing of a stimulus with the effective accommodative demand shown on the *x* axis. The blue disks show the measured convergence, converted to diopters assuming an interocular distance of 7 cm. The blue line in Figures 12C and 12D shows the stimulus gradient AC/A ratio as a function of the initial demand. This is computed by using the smoothed spline lines in Figures 12A and 12B to estimate the increase in ocular convergence when accommodative demand increases by 1 D from the initial demand shown in the *x* axis. The red line shows the same thing for ocular accommodation.

Both subjects show AC/A ratios of approximately 1 over most of their accommodative range. However, around the near point, AC/A ratio increases for both subjects. Consider subject MJL (Figures 12B and 12D). This observer has a maximum ocular power of around 6 D: beyond that point, his ocular power fails to increase in response to increasing demand. However, his ocular convergence not only continues to increase but from 6D to 8 D, it actually increases more steeply for each diopter of accommodative demand, resulting in a nearly fourfold increase in stimulus AC/A ratio. This result presumably represents the increase of accommodative effort as the observer strains to focus on the near stimulus, an effect qualitatively similar to that seen in the original model (Figure 11). However, this fourfold increase is still lower than plausible for the integrator gain. And for still closer distances, the stimulus AC/A ratio drops back to a value of around 1, closer to the predictions of our new model.

Suggestively, however, we do see very large increases in AC/A ratio when accommodation is paralyzed with homatropine to produce a temporary presbyopia. Then, efforts of accommodation are associated with large increases in AC gain (Christoferson & Ogle, 1956). The difference is that here, the range of accommodation is limited pharmacologically, in a way that cannot be known to the neural control system in advance. We present simulations for pharmacological cycloplegia below (Figures 14–17).

#### Cycloplegia: Hyperopic shift

As noted elsewhere in this article, the effect of a cycloplegic drug is modelled as a gain change in the oculomotor nerve signal (triangular gain block in Figures 1 and 4). Importantly, the cycloplegic gain reduction appears in the input to the physical plant, but not in the input to the virtual plant, reflecting the assumption that the brain is unaware of this gain change. The cycloplegic effect is described by the model parameter *Cycloplegia*, which ranges from 0 (no cycloplegia, oculomotor nerve signal is unchanged) to 1 (complete cycloplegia, oculomotor nerve signal to the physical plant is abolished). Adding a lower bound to the neural control signal, represented by the model parameter *NeuralMyopia*, enables the model to reproduce the hyperopic shift observed with cycloplegia. The original model behaved sensibly under cycloplegia, so this aspect of the model did not need fixing. We now demonstrate that our proposed new model also gives sensible results.

**Figure 13.**
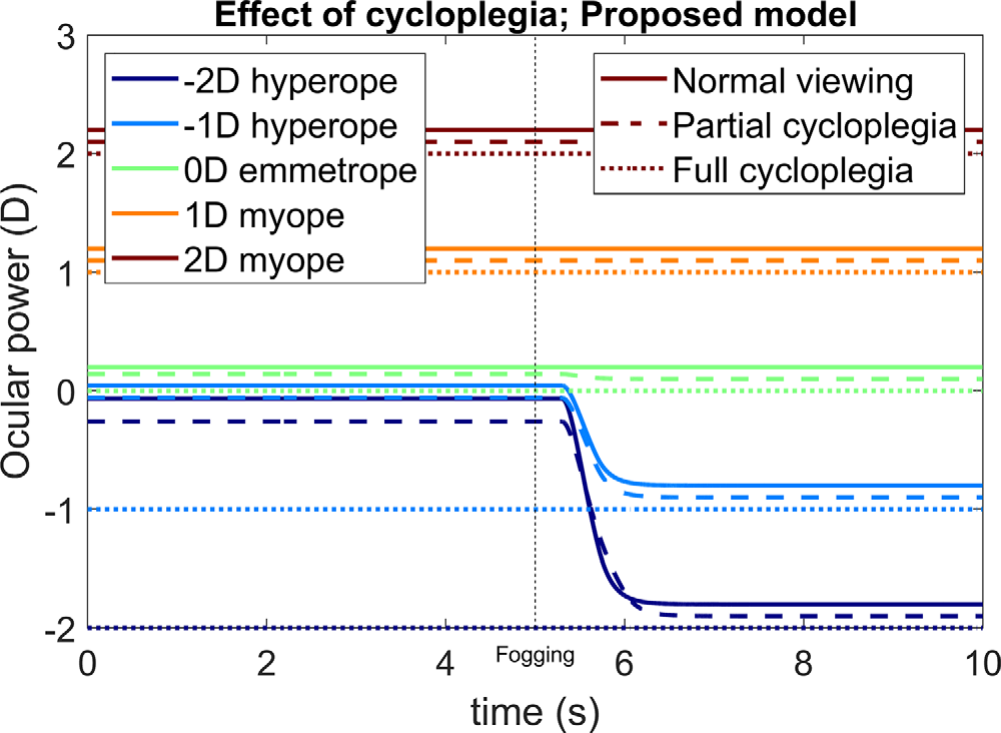
Time course of accommodation for observers with 5 different refractive errors (color legend) with normal viewing (solid line, model parameter cycloplegia = 0), with partial cycloplegia (dashed line, cycloplegia = 0.5) and with complete cycloplegia (dotted line, cycloplegia = 1). In each case, the observer is viewing a stimulus at infinity (0 D) and their accommodative range is 4 D. At time *t* = 5 seconds, a +3-D fogging lens is added. The model is that shown in Figure 5 (“original model”) with parameters specified in this legend and in Table 1. This figure was obtained with Matlab file PlotOcularPowerCycloplegia.m. Results with the original model are qualitatively similar for this example and are thus not shown in the article, although they can be generated using the Matlab file.

Figure 13 illustrates the hyperopic shift often seen under partial and complete cycloplegia. This figure shows the time course of accommodation for five different simulated observers with the refractive errors indicated in the color legend. Results are shown for normal viewing, partial cycloplegia and complete cycloplegia. For each observer, the value of *NeuralMyopia* is 0.2 D. The observers are viewing an object at infinity, initially with no external lens and then through a +3-D fogging lens applied at *t* = 5 seconds. With complete cycloplegia (dotted line, *Cycloplegia* = 1), the value of accommodation is exactly zero and so each observer's ocular power is simply their refractive error. In normal viewing with no cycloplegia (solid line, *Cycloplegia* = 0), the minimum value of accommodation is *NeuralMyopia* = 0.2 D. The emmetropic (0 D) and myopic (+1 D, +2 D) observers, therefore, end up with ocular power 0.2 D greater than their respective refractive errors; the addition of the plus lens at *t* = 5 seconds makes no difference because they were already relaxing accommodation as much as possible. The two hyperopic observers, however, initially (during the first 5 seconds) need to accommodate to bring the distant stimulus into focus. During this time, their ocular power is a little lower than that of the emmetrope, because it is set by the finite gain of the feedback loop (accommodation is already above the lower bound set by the minimum neural signal). When the +3-D lens is applied at *t* = 5 seconds, the two hyperopes relax accommodation and so their ocular power drops. The dashed lines show similar results for partial cycloplegia (*Cycloplegia* = 0.5). The key point is that, for each simulated observer, the ocular power with cycloplegia (dashed line) is lower than the ocular power measured with distance viewing through plus lenses (solid line for *t* > 5 seconds).

This confirms that placing a lower bound on the signal sent to the oculomotor nerve gives a realistic account of the effect of cycloplegia: cycloplegia produces a hyperopic shift, regardless of the observer's original refractive error, as observed empirically (Bagheri et al., 2018; Hiraoka et al., 2013; Yoo et al., 2017).

#### Cycloplegia: Increase in the stimulus AC/A ratio

**Figure 14.**
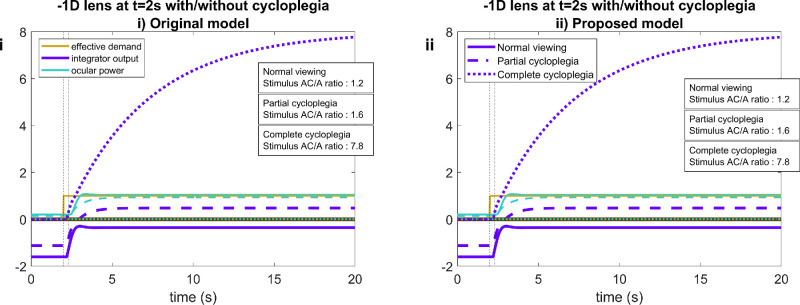
Large AC/A ratio observed with cycloplegia, for both the original and proposed model. Time-courses of signals in (**i**) the original and (**ii**) proposed model for an experiment in which an emmetropic observer views a distant stimulus, at 0 D, either with normal monocular viewing (solid lines) or with pharmacological cycloplegia (dashed lines). At time *t* = 2 seconds, a −1-D lens is inserted, raising the effective demand by 1 D. When the observer is cyclopleged, the resulting defocus cannot be corrected. Because this is not predicted by the accommodative control system's virtual model of the plant, the integrator output becomes very large. This in turn would lead to very large stimulus AC/A ratios. This figure was obtained with PlotACARatioCycloplegia.m.

Figure 14 confirms that our model still predicts large stimulus AC/A ratios with cycloplegia, as observed empirically (Christoferson & Ogle, 1956). Figure 14 shows the integrator output and ocular power for an emmetropic observer viewing a stimulus at 0 D, before and after the effective demand is increased by the insertion of a −1-D lens. Solid lines show the normal responses, and dashed/dotted lines responses when the observer has been partially or completely cyclopleged. In normal viewing (solid lines), ocular power is initially 0.2 D, reflecting neural myopia. The steady-state defocus is thus −0.2 D and the integrator is at −1.6 D. Following the insertion of the −1-D lens, the ocular power increases to 1 D and the integrator output rises to −0.4 D. This would imply a stimulus AC/A ratio of around 1.2 (the exact value depending on the AC gain and on the properties of the vergence control system).

The dashed line shows the situation for partial cycloplegia, with the model parameter *Cycloplegia* = 0.5. This decreases the gain of the system, but of course the virtual plant is unaware of the gain change so the internal estimates of accommodation and demand are erroneously high. The integrator output also increases as it strives to keep the stimulus in focus despite the lower gain. This reduction of gain thus increases the AC/A ratio. Finally, dotted lines show the situation for complete cycloplegia, where the feedback loop is severed and ocular power is at 0 D throughout. Following the insertion of the −1-D lens, the observer experiences sustained defocus of +1 D. Critically, this is not predicted by the observer's accommodative control system, which is expecting the lens to accommodate in response. Thus, the defocus is classed as “correctable” and is fed into the integrator, charging it up to saturation. The stimulus AC/A ratio thus increases by roughly the gain of the integrator relative to normal viewing, just as in the original model.

#### Cycloplegia: Demand–response curves

**Figure 15.**
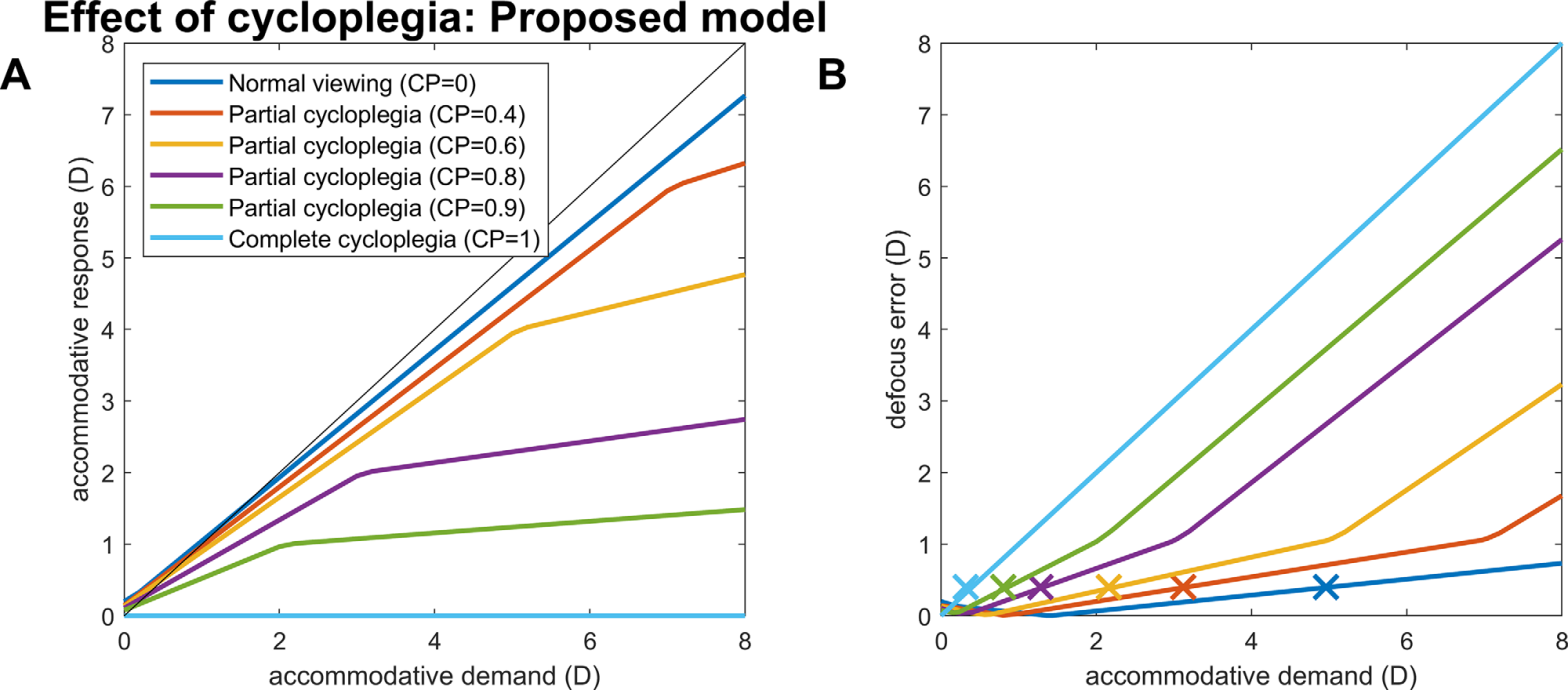
Demand–response curve in the presence of cycloplegia, for the proposed new model. (**A**) Steady-state accommodative response for the steady-state demand indicated on the x-axis, for the amount of cycloplegia indicated in the legend. (**B**) Magnitude of the steady-state defocus error (i.e., accommodation lag). Crosses x mark the subjective near point assuming a blur threshold of 0.3 D, that is, the highest demand for which steady-state defocus does not exceed 0.3 D. Model parameters are as in Table 1 with an accommodative range of 10 D and 0 D refractive error. This figure was obtained with PlotNearpointCycloplegia.m.

We now examine the effect of varying the amount of cycloplegia in more detail. Figure 15A shows the demand/response curve in the presence of different amounts of cycloplegia. As expected, the gradient of the line reduces as cycloplegia increases, becoming flat when cycloplegia is complete.

The curves for partial cycloplegia show a distinct “knee”: the gradient becomes much flatter for demands beyond a certain point. This is not observed in current models; there, the initial steep gradient persists throughout. See the Appendix for a detailed discussion of why this occurs.

Basically, the knee occurs because there are now two virtual plants in the internal feedback loop (see Figures 2 and 7). The realistic virtual plant has an upper saturation limit of the AR and is used to estimate demand, whereas the idealized virtual plant has an unlimited upper limit and is used to estimate correctable defocus for both accommodation and accommodative convergence. The knee occurs when the neural control signal exceeds AR. As cycloplegia increases, this point occurs at progressively lower demands, since cycloplegia increases the neural control signal required to elicit a given response. One can show mathematically that the slope is $\frac{G.CP^{'}}{1+G.CP^{'}}$ for demands less than *AR*(*CP*′ + *G*^−1^) and decreases to $\frac{G.CP^{'}}{1+G+G.CP^{'}}$ for greater demands, where CP′ = 1 − *Cycloplegia*, AR, and *G* is the gain of the integrator. In contrast, the original model predicts a slope of $\frac{G.CP^{'}}{1+G.CP^{'}}$ throughout. We are not aware of empirical data that would allow us to test which model gives a better account of human responses, but this is an interesting point for further study.

#### Cycloplegia: Reduction in near point

**Figure 16.**
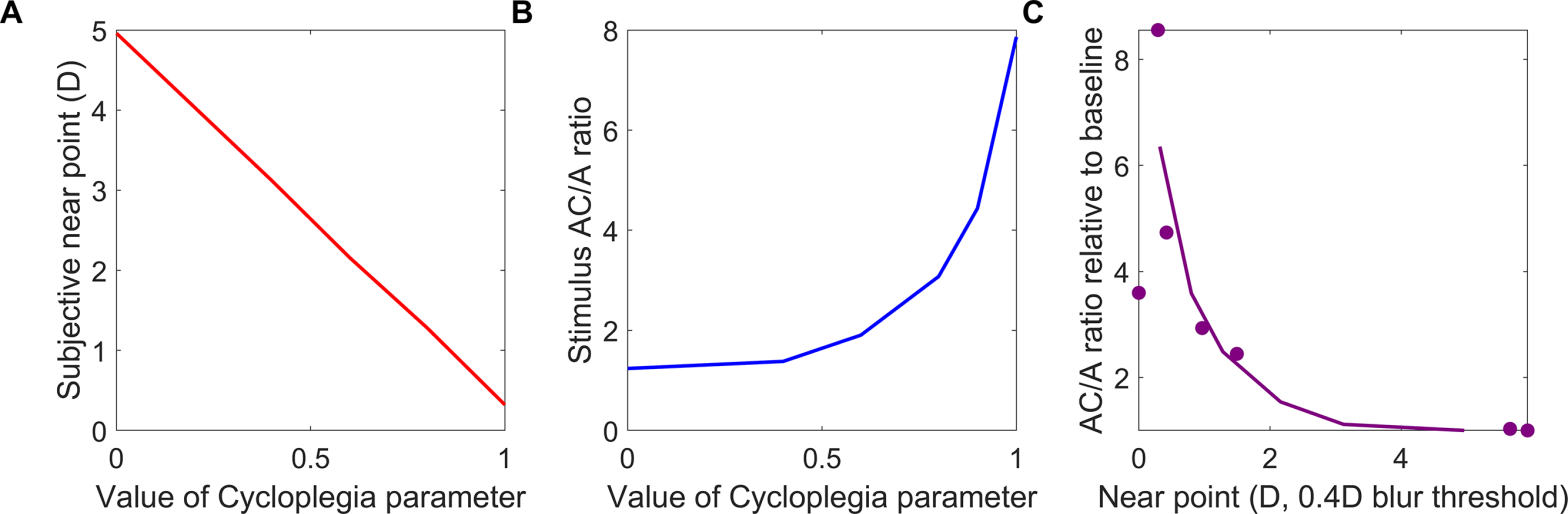
Subjective near point and stimulus AC/A ratios in the presence of cycloplegia. (**A**) Subjective near point (crosses in Figure 15B) as a function of the cycloplegia model parameter (where 0 indicates normal viewing and 1 complete cycloplegia). (**B**) Stimulus AC/A ratio, estimated as the total change in integrator output before vs after a −1-D lens is inserted while the model observer views a stimulus at 0 D (as in Figure 14), again as a function of cycloplegia. (**C**) Stimulus AC/A ratio relative to baseline (i.e., divided by the value measured with no cycloplegia) as a function of subjective near-point. Solid line: for the model observer shown in (A); dots: for the representative subject of Christoferson and Ogle (1956); data digitized from their Figure 4. Model parameters are as in Table 1 with an accommodative range of 10 D and 0 D refractive error. This figure was obtained with PlotNearpointCycloplegia.m.

**Figure 17.**
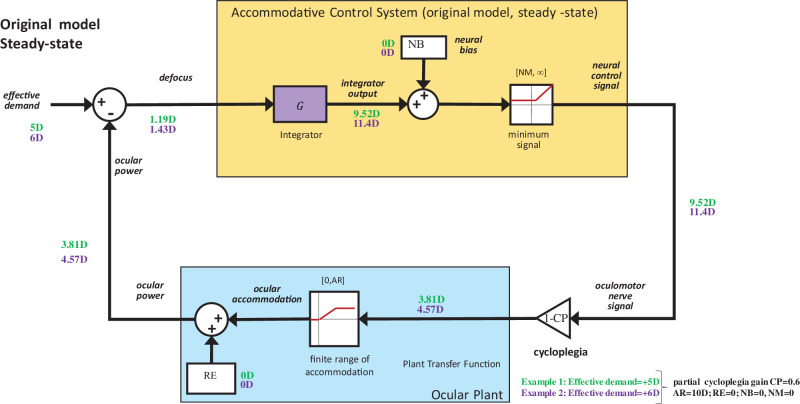
Simplified version of the original model, suitable only for obtaining its steady-state response. Note that, in the steady state, the predictive nature of the model is irrelevant. Small numbers show signal values for two examples, specified in the bottom right.

Figure 15B shows how we infer the subjective near point from the simulations shown in Figure 15A. The subjective near point is the distance where observers first notice a blur of the accommodative stimulus, and this is lower than the upper range of accommodation (AR). The colored traces in Figure 15B show the magnitude of the defocus error (the difference between the demand and response). The crosses *x* show the maximum demand where the defocus remains less than 0.3 D, taken to be the threshold for reporting perceptible blur. Figure 16A shows how the inferred subjective near point drops with increasing cycloplegia. Figure 16B shows the stimulus AC/A ratio, before the knee, estimated as in Figure 14: that is, while the model observer views a stimulus at infinity, we add in a −1-D lens and observe the change in integrator output, for different values of the *Cycloplegia* parameter. Figure 4 in Christoferson and Ogle (1956) similarly plots AC/A ratio and near point for their example observer as a function of time since they administered homatropine. We do not know the mapping from time to the value of the *Cycloplegia* parameter, but we can examine the agreement between model and data by plotting stimulus AC/A ratio (normalized to baseline, since this differs between observers) as a function of near point. This is done in Figure 16C. The solid line shows results for the model simulation; the dots are values for the example observer of Christoferson and Ogle (1956). Qualitatively, the agreement is good, with the AC/A ratio increasing first slowly, then steeply, as near point reduces. The main discrepancy is that Christoferson and Ogle (1956) found that the maximum AC/A ratio occurred after the minimum value of the near point (the highest symbol in Figure 16B is not the leftmost).

## Discussion

In this article, we have shown that conventional ways of incorporating refractive error into models of accommodative control predict unrealistically long response latencies after periods of defocus, whether these are due to refractive error, functional presbyopia, or to fogging with external lenses. This is a well-known problem within control theory, occurring when actuators with limited range are used with integrative control, and is known as integrator wind-up (Astrom & Rundqwist, 1989; Fertik & Ross, 1967; Krikelis & Barkas, 1984). To our knowledge, this problem has not been discussed before in the context of accommodative control, presumably because previous work has not examined the response of these models to stimuli with unattainable demand. In real life, however, such situations arise frequently owing to imperfectly corrected refractive error and/or functional presbyopia. It is thus important to produce models of accommodative control which behave correctly in these situations.

The contribution of this article is to argue that the blur-driven signal to accommodative control is not defocus error itself, but rather correctable defocus, defined as defocus, which could be removed by an ideal accommodative response. We thus adjust the predicted demand input to the controller such that the signal fed into the integrator is not the predicted future defocus itself, but only that component of predicted future defocus which is correctable by an idealized ocular plant, that is, the estimated future ideally correctable defocus (Figure 4)*.* This model is just slightly more sophisticated than existing predictive models of accommodative control.

An unusual feature of this model is the use of two separate virtual plants: a realistic one, incorporating the actual accommodative range, to estimate stimulus demand, and an idealized one to compute correctable defocus. Why would the brain develop two separate virtual plants? Perhaps because it is more efficient to perform the same computation locally than to transfer the information. Additionally, the outputs are used for different purposes, so it may be advantageous to develop separate virtual plants with different properties, as we now discuss.

The realistic virtual plant enables the brain to make realistic estimates of the stimulus distance from defocus, supporting the use of defocus as a depth cue (Held, Cooper, & Banks, 2012; Mather, 1996). This step assumes that the brain has been able to accurately estimate the eye's accommodative range. This process is not implausible, given that accommodative range changes on a timescale of years, and that predictive control already postulates that the control system has learnt a model of the ocular plant.

The idealized virtual plant is used to compute correctable defocus. The idealization is useful because, owing to presbyopia, all humans end up experiencing stimuli that are too close for them to accommodate on. Yet there remains value in generating a positive command signal in this situation, both to drive accommodation to the maximum possible and to assist convergence. However, an excessive command signal would both produce inappropriate convergence and delay the response to subsequent stimuli. The idealized virtual plant generates a positive signal in this situation, but ensures that it stays appropriate to the demand (despite the lack of accommodative response). In contrast, stimuli that are too far to accommodate on should never occur for an emmetropic observer. Commanding a negative accommodation is futile within the accommodation system and, if passed on to the vergence system, would produce disruptive divergence. Accordingly, our model avoids generating any signal in this situation. This accords with the clinical experience of author C.S. that myopes do not generally show accommodative divergence when a stimulus already beyond their far point is moved optically still further by the addition of plus lenses.

Although our model postulates that the control system learns the eye's finite accommodative range, it does not need to learn the eye's refractive error. Integrator wind-up is avoided whether or not the reason for the uncorrectable defocus could in theory be known to the brain (as in refractive error) or not (as with fogging by an externally applied lens).

We have also extended the model to handle pharmacological cycloplegia and demonstrate that it gives sensible results for both the subjective near point and the stimulus AC/A ratio in partial as well as complete cycloplegia. This has revealed further interesting nonlinear properties of our model, such as the prediction that with partial cycloplegia, the slope of the demand–response curve will not be reduced uniformly, but will be reduced more strongly for larger demands. This hypothesis seems reasonably plausible, but has not been tested.

Our postulated modification is fairly simple, plausible, and works well in a wide range of clinically relevant situations for accommodation. Whereas the original model showed unrealistically long latencies, we believe our proposed model behaves much more as clinicians would expect. Although we have not simulated full accommodation/vergence models here, we also believe that our modification also makes the output of the accommodative integrator more suitable for use as the accommodative convergence signal in such models.

However, many other approaches are undoubtedly possible. One limitation of our model is that we assume defocus is the sole error function minimized by the system. There could also be other inputs, for example, voluntary or involuntary accommodative effort driven by nonoptical cues to proximity. For example, the increase in stimulus AC/A ratio reported by Alpern et al. (1959) around the end of the accommodative range might reflect an additional voluntary accommodative effort, after the normal involuntary response driven by defocus failed to clear the retinal image.

A similar simplification is that we assume that the predicted future ideal accommodation and attainable demand have no upper bound. More realistically, an upper bound such as the amplitude of accommodation at birth might be used to estimate the ideal attainable bound (D_max_). This factor could perhaps be quantified from empirical measures of the upper limit to accommodative convergence (AC_max_) in absolute presbyopes, assuming that the gain of AC (AC/A ratio) is fixed where D_max_ = AC_max_/(gain of AC).

Our discovery of this defect in current models has highlighted a need for empirical data on how ocular accommodation behaves during and after exposure to stimuli beyond the accommodative range. Our model makes specific predictions, which we believe are more accurate than those of current models, but more data in this regime might well reveal a need for further modifications.

We also cannot know whether our model is at all an accurate representation of the underlying neurophysiology (McDougal & Gamlin, 2015). To our knowledge, none of the existing literature can speak to this, since no studies have examined the response to stimuli out of the response range. For example, Zhang, Mays, and Gamlin (1992) identified near response neurons in the midbrain whose firing rate correlates with ocular accommodation for stimuli within the response range of the neuron. In our model, these might correspond with the motor signal itself or with the estimated future demand, or to the estimated future limited demand, because all these correlate with ocular accommodation for steady-state stimuli. Our model makes different predictions about the behavior of these three for stimuli out of the response range, but these were not tested. Zhang and Gamlin (1998) found far response neurons in the posterior interposed nucleus of the cerebellum, whose firing rate increased as accommodation decreased. Microstimulation of this area often elicited decreases in accommodation if accommodation was near, but had no effect if accommodation was already far. Again, this finding is consistent with our model, but hardly diagnostic. For example, these far response neurons might represent the output of the integrator with a sign inversion; microstimulation would then request a decrease in accommodation, but the saturation block in the motor signal would explain the lack of response when accommodation is already far. Much more detailed neurophysiology, involving stimuli both within and beyond the range of accommodation, will be needed to elucidate whether this model really captures neural computations. For such experiments, it will be critical to present both correctable and uncorrectable defocus. Our model predicts that at the sensory end of the neural pathway, the response to defocus of a given magnitude will be the same regardless of whether the defocus is correctable or not. Closer to the motor output, one should see a clear difference, with uncorrectable defocus not eliciting a signal in the oculomotor nerve.

The model also suggests interesting lines of inquiry regarding myopia progression. Myopic eyes tend to become more myopic over time, whereas hyperopic eyes are much more stable (Mäntyjärvi, 1985). Our model postulates different anti–wind-up solutions for the negative defocus produced by myopia and the positive defocus produced by hyperopia. In our model, positive defocus produces an initial accommodative signal that we postulate is relayed by the accommodative convergence cross-link to produce a convergence response. Thus, hyperopia produces an esophoria error. However, in our model, the negative defocus error produced by the stimuli beyond the far point is not fed forward either to the accommodative integrator or to the accommodative vergence system (achieved by the saturation block labelled “not too far” in Figure 6). Thus, myopia produces no exophoria error in this situation, although it does for stimuli that are closer than the far point. Could phoria error act as a penalty, discouraging the increase of refractive error? If so, the asymmetric is phoria produced by hyperopia vs myopia could contribute to the difference in progression rates.

Whether or not our model proves to be a good account of the computations actually performed in the brain, it provides the most accurate account to date of real accommodative responses in a range of clinically relevant situations. Matlab Simulink models and code are provided online which enable researchers to estimate the response time course of human observers with realistic clinical conditions to arbitrary accommodative stimuli.

## Figures and Tables

**Figure 18.**
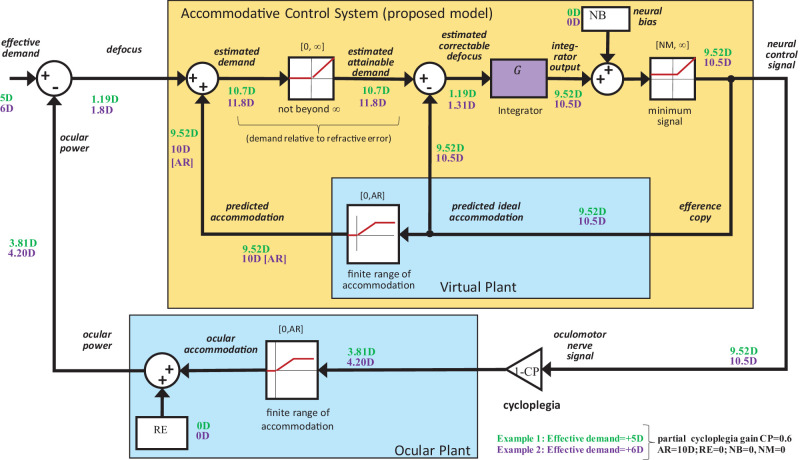
Simplified version of our proposed new model, suitable only for obtaining its steady-state response. The small numbers show the steady-state signal values under partial cycloplegia (CP = 0.6) for effective demands of 5 D [green] and 6 D [purple numbers]). The accommodative range is an AR of 10 D and the gain od 8. The neural bias is set to zero for simplicity (NB = 0) and the neural myopia is assumed to be small enough that its value does not affect the results. With the specified values, the “knee” is 5.25 D, so the two example demands are on either side of this value.

## Appendix

### Understanding the slope change in the demand/response curve with cycloplegia

For interested readers, we now go through why the change in slope occurs for our new model (Figures 2 and 7), and why it does not for the original model (Figure 5). To explain this, it will be helpful to redraw both models in a simplified form suitable for understanding their steady-state response. For this, we can omit all delays, such as those owing to the sensorimotor latency, because a steady-state signal is unaffected by a delay, and we can replace all transfer functions by their steady-state values. Thus, the physical and virtual plant transfer functions disappear altogether, as does the demand extrapolator, because, for all these, in the steady state, the output of the block equals the input to the block and so the block has no effect. The integrator, meanwhile, is represented only by its gain *G*.

In the original model, after these modifications a further simplification becomes possible. Look at the accommodative control system in the original model (Figure 5). In the steady state, the predicted past and estimated future demands are equal, as are the predicted past and future accommodation signals. Effectively, therefore, the signal from the virtual plant is added on and then subtracted immediately. We can, thus, omit the virtual plant altogether without changing the model's behavior. This simplified steady-state representation of the model is shown in Figure 17. The predictive nature of the full model (Figure 5) is not relevant in this reduced steady-state version. Effectively, the control system acts directly on the optical defocus (retinal error signal), without reconstructing estimated demand. It is now easy to see that, until accommodation reaches its maximum value, *AccomRange*, cycloplegia acts purely to reduce the closed-loop gain of the system. The slope of the demand–response curve is reduced, but there is no “knee.”

The small green and purple numbers work through two simple examples, both with partial cycloplegia (=0.6) and for demands of 5 D and 6 D. The reduced cycloplegic gain reduces the ocular power for a given demand and, thus, increases defocus. The neural control signal (output of the neural integrator) is thus much larger than it would normally be to achieve the same accommodation: the system is having to “work harder” to achieve even these lower responses. Note that in both examples, the ratio of response to demand is the same, 0.76, reflecting the constant slope of the demand/response curve.

Figure 18 shows a similarly simplified version of our new model, again suitable only for modelling the steady state. However, note that now the virtual plant cannot be removed. This is because in our new model there are effectively two virtual plants: one incorporating the finite accommodative range of the physical plant and one not limited in this way. The predicted accommodation signal is the response of the amplitude limited virtual plant, and the predicted ideal accommodation signal is the response of the unlimited virtual plant. Estimated demand is computed by adding predicted accommodation to defocus, and the estimated correctable defocus is computed by subtracting the predicted ideal accommodation from the estimated attainable demand. Thus, the estimated demand is limited by the amplitude-limited virtual plant and the estimated correctable defocus by the unlimited virtual plant. When the neural control signal (output of the neural integrator that drives the plants) does not exceed the accommodative range of the physical plant, then the predicted ideal accommodation and predicted accommodation are equal. When equal, their effects cancel when they are added and subtracted when computing estimated demand and estimated correctable defocus, respectively. Usually, the effective demand (input) is positive relative to refraction, and the estimated attainable demand is equal to the estimated demand. Under these conditions, the estimated correctable defocus equals the optical defocus.

Once the neural control signal exceeds the accommodative range, the predicted accommodation and predicted ideal accommodation will not be equal and, thus, will not cancel when added to the defocus and subtracted from the estimated attainable demand, respectively. Additionally, although not relevant here, in general the estimated attainable demand might not be the same as the estimated demand, which could further change results. Consider the example where the neural control signal exceeds the accommodative range of the physical plant. In this example, it is clear from Figure 18 that the amplitude-limited predicted accommodation signal will be less than the unlimited predicted ideal accommodation. This means that, for sufficiently large effective demands, the estimated correctable defocus will be less than the optical defocus, whereas for smaller demands they are equal. This effect is what causes the change in slope of the demand/response curve (Figure 15). This illustrates an interesting feature of our proposed new model; namely, its predictive nature remains relevant even in the steady state.

To go through this in detail, the small numbers in Figure 18 show steady-state values for two different demands, 5 D and 6 D, with partial cycloplegia. The accommodative range is 10 D and cycloplegia is 0.6; thus, the change in slope occurs at 5.25 D. The 5-D demand (green numbers) is below this point, so our new model responds exactly the same as the original model (compare green numbers in Figures 17 vs. 18). However, now consider the situation for the 6-D demand (purple numbers). Here, the accommodation under partial cycloplegia is 4.2 D. Because of the cycloplegic gain reduction, to achieve this value, accommodation requires a neural control signal of 10.5 D, beyond the range of accommodation (10 D). The brain thus estimates the attainable demand as 11.8 D (10-D predicted accommodation, estimated by the amplitude-limited virtual plant, plus the 1.8-D defocus). This is actually slightly higher than the 11.4-D estimated for the 6-D demand the original model (Figure 17), owing to the higher optical defocus in our model. However, to compute the estimated correctable defocus, our model then subtracts the predicted ideal accommodation of 10.5 D, effectively the signal from a second virtual plant with unlimited amplitude. Because the 10.5-D signal subtracted from this unlimited virtual plant is larger than the 10-D signal originally added on from the limited virtual plant, the estimated correctable defocus signal, 1.3 D, ends up both being lower than the actual defocus, 1.8 D, and lower than the defocus fed into the integrator in the original model, 1.43 D (Figure 17). Thus, the effective gain of the system is now reduced even more than implied by the cycloplegic gain reduction. This nonlinear effect accounts for the change in slope of the demand/response curve (Figure 15): from a 0.76-D change in response for each 1-D change in demand for stimuli further than 5.25 D, to 0.26 D change in response per 1-D change in demand for nearer stimuli.
